# Recent developments using malononitrile in ultrasound-assisted multicomponent synthesis of heterocycles

**DOI:** 10.1016/j.ultsonch.2023.106741

**Published:** 2023-12-23

**Authors:** Ramin Javahershenas, Sahand Nikzat

**Affiliations:** a Department of Organic Chemistry, Faculty of Chemistry, Urmia University, Urmia, Iran; b Chemical Physics Theory Group, Department of Chemistry, University of Toronto, Toronto, Ontario M5S 3H6, Canada

**Keywords:** Malononitrile, Ultrasound, Sonochemistry, Heterocycles, Multicomponent reactions, Synthesis

## Abstract

Ultrasonic irradiation serves as a vigorous and environmentally sustainable approach for augmenting multicomponent reactions (MCRs), offering benefits such as thermal enhancement, agitation, and activation, among others. Malononitrile emerges as a versatile reagent in this context, participating in a myriad of MCRs to produce structurally diverse heterocyclic frameworks. This review encapsulates the critical role of malononitrile in the sonochemical multicomponent synthesis of these heterocyclic structures. The paper further delves into the biochemical and pharmacological implications of these heterocycles, elucidating their reaction mechanisms as well as delineating the method's scope and limitations. We furnish an overview of the merits and challenges inherent to this synthetic approach and offer insights for potential avenues in future research.

## Highlights

• Malononitrile.
• Ultrasound.
• Sonochemistry.
• Heterocycles.
• Multicomponent reactions.
• Synthesis.

## Graphical abstract

**Figure:**
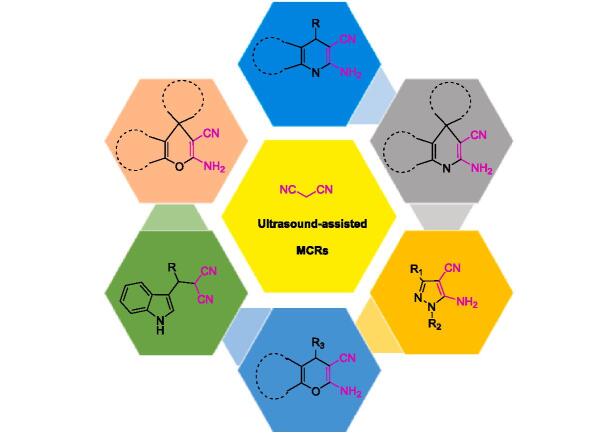

## 1 Introduction

Heterocyclic compounds are ubiquitous in nature and constitute the foundational structure for an extensive array of biologically active molecules and medicinal compounds, encompassing antibiotics, antiviral agents, and anticancer drugs [1]. Numerous natural products encapsulate these compounds, accentuating the imperative for chemists to devise synthetic methodologies that are both efficacious and environmentally benign [2]. The domain of medicinal chemistry and drug discovery is heavily predicated on heterocycles, which are distinctive organic compounds characterized by at least one ring structure devoid of carbon atoms [3]. Acquiring proficiency in the synthesis of assorted heterocyclic scaffolds is pivotal for the exploration and development of novel therapeutic agents [4]. Historically, the genesis of heterocycles has entailed a multi-step endeavor, encompassing myriad reaction stages and purification procedures. These conventional methodologies frequently encounter challenges such as diminished productivity, protracted reaction durations, and the emergence of byproducts. In cognizance of these challenges, recent scholarly endeavors have been oriented toward the formulation of more sustainable and streamlined synthetic methodologies [5], [6].

The field of organic synthesis has undergone a paradigm shift with the advent of multicomponent reactions (MCRs). Distinct from conventional reactions, which primarily entail the union of two reactive entities, MCRs orchestrate the concurrent reaction of three or more starting materials, culminating in a singular complex product in a lone step [7]. The resultant product encapsulates most, or at times all, of the atoms from the starting materials, epitomizing the efficiency and atom economy of MCRs. Typically, MCRs navigate through a cascade of intermediary steps where diverse functional groups engage in reactions in a predefined sequence [8]. Based on the nature of reactants, MCRs are segregated into various classes, inclusive of carbonyl compounds, isocyanides, organoboron compounds, free radicals, and metal catalysts [9]. The application spectrum of MCRs is broad in organic synthesis, especially in the quest and formulation of new drugs and bioactive molecules. Additionally, MCRs pave the way for the generation of combinatorial libraries encompassing potential drug candidates, which can be subjected to screening for biological efficacy. The prowess of MCRs to amalgamate multiple reactants in a singular stride confers myriad advantages including enhanced efficiency, atom economy, broadened reactant diversity, elevated complexity generation, diminished waste output, and an expanded chemical space diversity [10], [11].

Ultrasound synthesis emerges as a cutting-edge technology, leveraging high-frequency sound waves to engender and manipulate materials at microscopic scales. Sound waves, transcending the frequency range of human auditory perception, are generated to elicit diverse effects. This avant-garde technique capitalizes on the prowess of sound waves to manipulate and metamorphose substances, thereby augmenting efficiency, accuracy, and precision [12]. It has cast a transformative impact across myriad industries, encompassing medicine, electronics, and materials science. Also labeled as sonochemistry, ultrasound synthesis is a flourishing domain within chemistry, deploying high-frequency sound waves to expedite chemical reactions [13]. This modality has garnered substantial attention of late, courtesy of its manifold advantages. Several refinements have been introduced to these processes, such as amplification of reaction rates, curbing of energy expenditure, enhancement of selectivity, adoption of greener processes, and mitigation of waste generation [14]. A broad spectrum of organic reactions has been explored employing ultrasound synthesis in arenas like multicomponent reactions, organic synthesis, nanoparticle synthesis, and materials science for conjuring complex molecules. By wielding the effectiveness of ultrasound, scientists and chemists can attain meticulous control over the composition, structure, and attributes of materials, propelling forward strides in drug delivery systems, electronic gadgets, and advanced materials [15]. Ultrasound synthesis is amenable to a variety of reactions, including but not limited to condensation, addition, substitution, oxidation, reduction, polymerization, and multicomponent reactions. Additionally, ultrasound synthesis can be harmoniously melded with other techniques like microwave irradiation, photochemistry, electrochemistry, and sonochemistry, opening up a vista of possibilities in synthetic endeavors [16].

Ultrasound irradiation stands as an influential and environmentally amicable technique capable of augmenting multicomponent reactions (MCRs) through furnishing heat, agitation, activation, or synergism [17], [18]. The assimilation of ultrasound-assisted methodologies within multicomponent syntheses has, of late, piqued interest among the chemical scholarly circles. Ultrasonic waves expedite reactions by enabling molecular diffusion and mass transference among reactants and catalytic entities [19]. Moreover, these ultrasound-facilitated processes typically yield enhanced product outputs, abbreviated reaction durations, and diminished environmental ramifications when juxtaposed with conventional methodologies [20].

Malononitrile, a pivotal constituent in numerous chemical reactions, has ascended to prominence as an indispensable moiety, attributed to its highly reactive α-carbon – a characteristic fortified by the resonance stabilization bestowed by the adjacent cyano groups. Its propensity for engaging in a varied spectrum of reactions, including addition-substitution sequences, Michael additions, Knoevenagel condensations, Strecker syntheses, and Diels-Alder reactions, further underscores its significance [21], [22], [23]. By liaising with multiple electrophiles and nucleophiles, these varied reactivity paradigms facilitate the edification of heterocyclic scaffolds. These scaffolds comprise aldehydes, ketones, isocyanides, amines, and ylides, particularly within the ambit of ultrasound-assisted multicomponent reactions, thereby broadening the scope of chemical synthesis and diversification [24], [25]. Harnessing this approach, researchers have adeptly accessed an expansive class of biologically active molecules, encompassing pharmaceuticals, agrochemicals, and materials. These substances have promising uses in drug discovery and development since they display a diverse range of pharmacological activities, thus enhancing the synthesis of different heterocyclic frameworks [26], [27].

In this review, we reviewed recent progress in the multicomponent reactions [28], [29], [30], [31], [32] and summarize recent advances in the multicomponent sonochemical synthesis of heterocycles using malononitrile as a pivotal component. Because these heterocycles are applied to biology and pharmaceuticals, the mechanism of reaction, the scope and limitations of these transformations will be discussed. We also outline the benefits and challenges of this method and provide some perspectives for future research. This article discusses mainly malononitrile as a key molecule in ultrasound synthesizing heterocyclic compounds reported in the literature from 2015 to 2023.

## 2 Chemistry of malononitrile

Malononitrile, an organic compound characterized by the molecular formula CH_2_(CN)_2_, encompasses dual cyano functionalities (–CN) adjoining a central methylene consortium (–CH_2_-). This compound manifests as a colorless or white solid, albeit with a propensity to adopt yellow or brown hues over time. It exhibits solubility in a series of solvents including water, ethanol, and ethyl acetate [33]. Structurally, the molecule is planar, adhering to C2v symmetry. Delving into its chemistry unveils malononitrile as a versatile edifice for orchestrating the synthesis of a broad spectrum of organic compounds. Its distinctive architecture facilitates a multifaceted reactivity, thereby facilitating the genesis of various heterocyclic frameworks. This unique reactivity engenders the formation of multiple bonds and the inauguration of a varied set of functional groups in a single step. Malononitrile can undergo nucleophilic addition reactions at both cyano domains, alongside condensation reactions with carbonyl compounds, culminating in the formation of α,β-unsaturated nitriles [34].

Malononitrile is extensively used in organic synthesis as a versatile building block for many different useful compounds in chemistry. It has many interesting properties and reactions that make it appropriate for a myriad of purposes. Some applications of malononitrile include:

• In MCRs, Knoevenagel condensation is a common mechanism for malononitrile formation. The reaction of malononitrile with either an aldehyde or a ketone in the presence of a base results in an α,β-unsaturated nitrile. The base can be an organic amine, a metal salt, or a metal oxide. The mechanism involves the deprotonation of malononitrile by the base to form a carbanion, which then attacks the carbonyl group of the ketone or aldehyde. The resulting intermediate then loses a molecule of water to form a double bond and regenerate the base [35].
• Another conventional mechanism of malononitrile in MCRs is the Michael addition. To create a 1,4-dicarbonyl or 1,4-dinitrile compound, malononitrile reacts with a carbonyl in the presence of a base to produce an unsaturated carbonyl or nitroalkene. The base can be an organic amine, a metal salt, or a metal oxide. The mechanism involves the deprotonation of malononitrile by the base to form a carbanion, which then adds to the β-carbon of the unsaturated compound. The resulting intermediate then undergoes tautomerization to form the final product and regenerate the base [36].
• The Gewald reaction is a chemical reaction that involves the formation of a thiophene ring from malononitrile, a ketone or an aldehyde, elemental sulfur, and a base. Thiophenes are heterocyclic compounds with various biological and industrial applications [37].
• The synthesis of donor–acceptor heterocyclic compounds, which have potential uses in molecular electronic devices and data storage. Compounds with these structures contain an electron-donating group and an electron-withdrawing group connected by a conjugated bridge.
• The synthesis of photo-cross-linkable liquid crystalline polymers, which have optical and mechanical properties that can be controlled by light. These polymers are made from benzylidenemalononitrile derivatives that can form covalent bonds when exposed to ultraviolet radiation [38].
• The synthesis of nonlinear optical (NLO) compounds, characterized by their capacity to modulate their refractive index or polarization according to electric field or light intensity fluctuations, represents a noteworthy domain of synthesis. These compounds are indispensable in fields such as telecommunications and optical information processing due to their unique optical attributes. A prime instance of an NLO compound, derived from the utilization of malononitrile, is (E)-2-(3-(4-aminostyryl)-5,5-dimethylcyclohex-2-enylidene) malononitrile. This compound highlights the potential of malononitrile as a foundational entity in the synthesis of functionally diverse and application-oriented molecules [39].

## 3 Sonochemistry

**Fig. 1:**
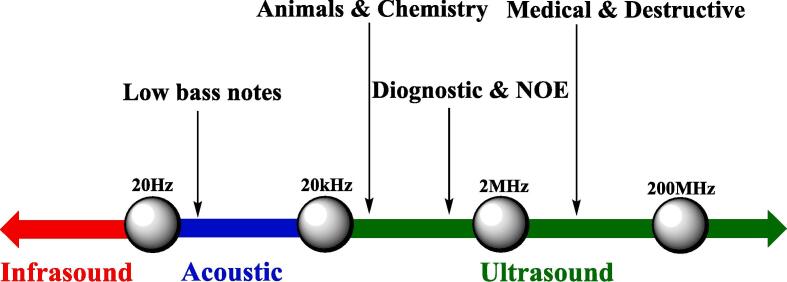
Sonic frequencies.

The relentless endeavor by chemists to craft and synthesize novel heterocyclic compounds, catered towards specific applications across pharmaceutical, food, and health sectors, has garnered substantial interest [40]. The genesis of these compounds necessitates the disintegration of bonds within the starting materials and the subsequent formation of new bonds, demanding a plethora of conditions to be met—appropriate reaction medium, temperature, solvent, and apt reactants [41]. The process of bond dissociation and reformation is energy-intensive, with various methodologies like thermal heating, microwave irradiation, and ultrasonic waves being employed to furnish the requisite energy, contingent on the nature of the starting materials, reaction conditions, and anticipated products [42]. Owing to its inherent characteristics, the ultrasonic process, employing sound waves, is regarded as an exemplar of green energy. The utilization of ultrasound, characterized by sound waves with frequencies exceeding 20 KHz and being inaudible to human auditory faculties (Fig. 1), is witnessing an uptick owing to its myriad advantages [43], [44].

**Fig. 2:**
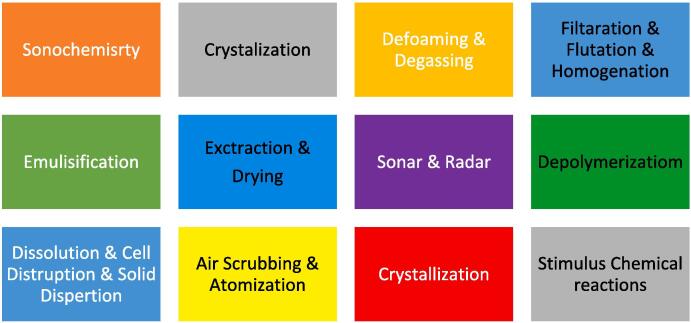
General application of ultrasound.

**Fig. 3:**
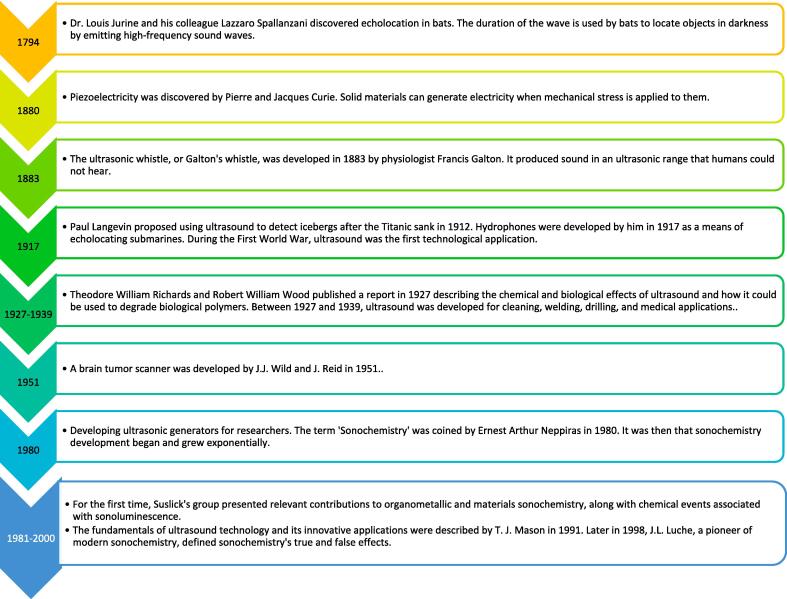
Historical background of ultrasound.

**Fig. 4:**
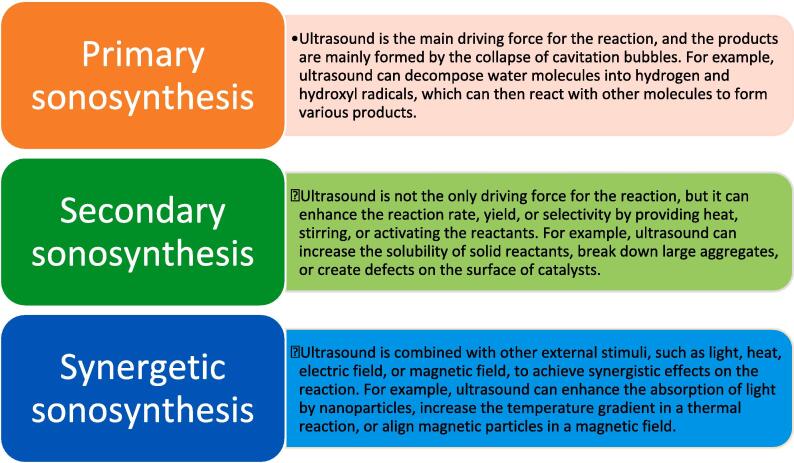
Classification of sonosynthesis.

**Fig. 5:**
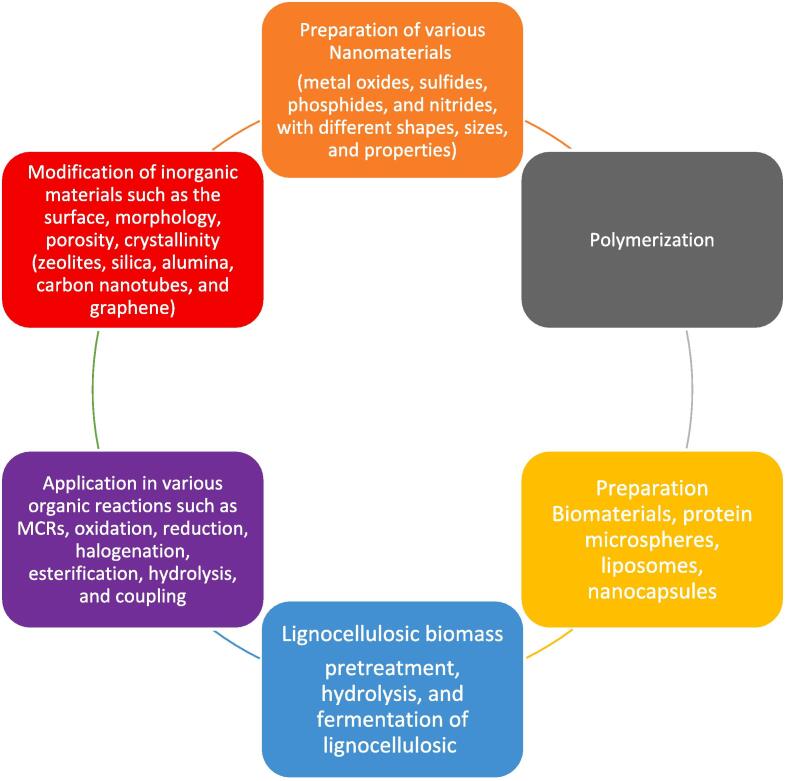
Some applications of sonosynthesis.

**Fig. 6:**
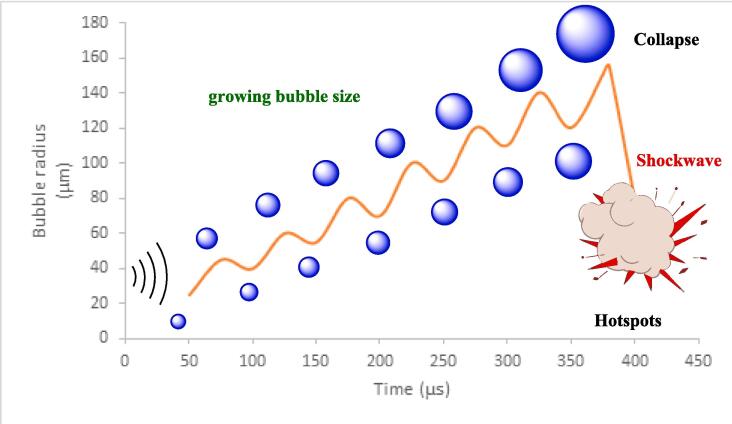
Schematic of acoustic cavitation.

Despite this, ultrasound has multiple practical applications in daily life, including sonar technology for navigation and echography for medical purposes (Fig. 2) [35], [36], [37], [38], [39], [40], [41], [42], [43], [44], [26]. Fig. 3, Fig. 4, Fig. 5, Fig. 6.

Sonosynthesis can be classified into three types depending on the role of ultrasound in the reaction [26], [45], [46], [47], [48]:

Sonosynthesis represents a specialized field focused on the investigation of ultrasound's influence on chemical reactions and processes. Employing ultrasonic irradiation, this technique facilitates the synthesis of a wide range of materials, including but not limited to nanoparticles, polymers, and heterocyclic compounds [49], [50], [51], [52].

## 4 The phenomenon of acoustic cavitation

Ultrasound irradiation is a technique that uses high-frequency sound waves to create bubbles in a liquid medium. These bubbles grow and collapse rapidly, producing intense local heat and pressure. This phenomenon is called acoustic cavitation, and it can initiate or accelerate chemical reactions in a liquid [53]. Localized hotspots are generated as a result of the violent collapse of these bubbles, which have temperatures exceeding high temperatures (4500–5000 K) and very high pressures (1000 atm). These extreme conditions created by ultrasonic waves can significantly enhance the rate of chemical reactions [54].

The substantial thermal and mechanical energy generated during the cavitation process possesses the capability to cleave chemical bonds, expedite reaction kinetics, and engender the creation of novel molecular structures. Moreover, the implosive collapse of cavitation bubbles induces rapid mixing, thereby ensuring an efficient mass transfer and a homogeneous dispersion of reactants, which serves to augment the overall efficiency of the chemical reaction [55].

Sonochemistry, alternatively known as sonosynthesis, entails the utilization of ultrasonic irradiation as a catalyst for material synthesis, aiming to scrutinize the impact of shockwaves generated by localized pressure fluctuations and the reactive species produced during the thermal decomposition of molecules in the vicinities of cavitation bubbles [56].A salient advantage of this ultrasound-facilitated approach is the pronounced acceleration of reaction kinetics, resulting in expedited reaction rates compared to traditional synthesis methodologies, thereby minimizing the requisite reaction time. Beyond kinetic acceleration, ultrasonic waves enhance the efficiency of chemical reactions through the promotion of effective mass transfer and the assurance of homogeneous reactant distribution. The swift mixing instigated by the implosive collapse of cavitation bubbles fosters optimal reactant contact, thereby facilitating more proficient molecular interactions and culminating in elevated reaction yields [56].

Acoustic cavitation constitutes a compelling physical phenomenon manifesting upon the exposure of a liquid medium to high-intensity acoustic waves, leading to the inception, expansion, and eventual implosion of vapor-encapsulated microcavities [40], [41], [42]. The ensuing effects of this process on subjected substances and materials can range from beneficial to deleterious. This evaluation aims to furnish a comprehensive examination of the attributes of acoustic cavitation, variables modulating its occurrence, requisite conditions facilitating its induction, as well as its manifold practical applications and conceivable negative repercussions [43], [44].

To begin with, a fundamental grasp of the underlying dynamics of acoustic cavitation is imperative. When a liquid medium is exposed to acoustic waves of sufficiently high frequency and intensity, oscillations in pressure ensue. These oscillatory pressure variations engender localized tension zones within the liquid, where microbubbles materialize due to the entrapment of dissolved gases or other particulate impurities [26], [45]. Subject to ongoing pressure cycles emanating from the acoustic waves, these microbubbles experience growth and may subsequently amalgamate with neighboring bubbles to constitute larger cavities. Upon reaching a critical dimension, these cavities undergo abrupt, violent collapse due to the differential pressures, culminating in the release of localized bursts of energy. This energy discharge gives rise to various phenomena, including but not limited to, shockwave propagation, jet formations, and extreme localized temperature and pressure conditions [46], [47].

Factors that modulate the incidence and dynamics of acoustic cavitation include the frequency and intensity of the acoustic waves, in conjunction with the intrinsic properties of the liquid subjected to sonication. High-intensity sound waves amplify the propensity for cavity initiation, as they induce substantial pressure fluctuations within the liquid medium [48], [49], [50]. Additionally, sound waves of lower frequency generally promote bubble enlargement more efficaciously compared to their high-frequency counterparts, attributable to their extended wavelength and enhanced penetration depth. Liquid properties, such as viscosity, also significantly dictate the likelihood of cavitation; for instance, a fluid with lower viscosity exhibits a higher susceptibility to undergo acoustic cavitation relative to one characterized by higher viscosity [51], [52], [53].

Prior to the onset of acoustic cavitation, particular preconditions must be fulfilled. Firstly, an initial nucleation site within the liquid medium is requisite for the formation of microbubbles; such sites may be constituted by pre-existing gaseous inclusions or particulate impurities. Secondly, an adequate pressure differential, exceeding a certain threshold, is essential to instigate the genesis and subsequent expansion of the bubble. Lastly, the bubble must attain a critical dimension to facilitate its implosive collapse, thereby generating the characteristic release of localized energy [54], [55].

Acoustic cavitation boasts a plethora of practical applications, spanning diverse sectors such as medicine, engineering, and environmental sciences. A particularly promising realm for its deployment is sonochemistry, where acoustic cavitation serves to catalyze or expedite chemical reactions by the localized extreme temperatures and pressures it generates. Within this domain, one widely acclaimed application entails the sonochemical degradation of contaminants in wastewater treatment processes [56], [57].

Another noteworthy application in the medical field is the use of High-Intensity Focused Ultrasound (HIFU). This technique capitalizes on the principles of acoustic cavitation to facilitate non-invasive interventions, including but not limited to, tumor ablation and lithotripsy. HIFU operates through the meticulous focusing of ultrasound waves onto designated tissue regions, thereby inducing localized cavitation within the target zone. This selective cavitation culminates in the destruction of target cells while concomitantly preserving the integrity of adjacent healthy tissues [58].

To summarize, acoustic waves have inaugurated a paradigm shift in the realm of chemical reactions by proffering a multifaceted and efficacious approach to reaction facilitation. Owing to their capacity to expedite reaction kinetics, refine selectivity, and optimize mass transfer, ultrasound-assisted chemical processes present substantial promise for driving innovations across diverse research fields and industrial applications.

Synthetic molecules can be classified according to their structural characteristics, which facilitate their analysis into four types. These include *N*-heterocyclic compounds, *O*-heterocyclic compounds, *S*-heterocyclic compounds, spiro compounds, and miscellaneous compounds. Formation of final products underlies the organization of these categories. Therefore, this approach presents a simplified and effortless manner of investigating synthetic molecules.

## 5 N-Heterocyclic compounds

### 5.1 1,4-dihydropyridine derivatives

**Scheme 1:**
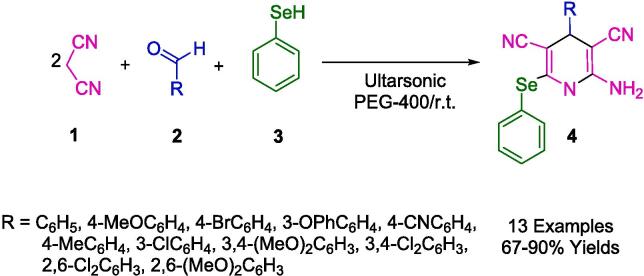
Synthesis of highly functionalized seleno-1,4-dihydropyridines.

Among the various and numerous chemical compounds that have a wide range of medicinal and biological activities, chemists can mention organoseleniums, which are of special interest. There are numerous types of these substances, such as antioxidants, enzyme modulators, antimicrobials, antitumors, antiviral, antihypertensive agents, cytokine inducers, and anticancer agents. For the preparation of 2-amino selenopyridine derivatives (**4**) by ultrasound-assisted multicomponent reactions using phenylselenol (**3**), malononitrile (**1**), and a series of substituted aldehydes (**2**), Choudhury *et al.* published an ultrasound-assisted reaction in polyethylene glycol (PEG-400) (Scheme 1). The catalyst-free reaction was performed using an ultrasonic bath [59].

**Scheme 2:**
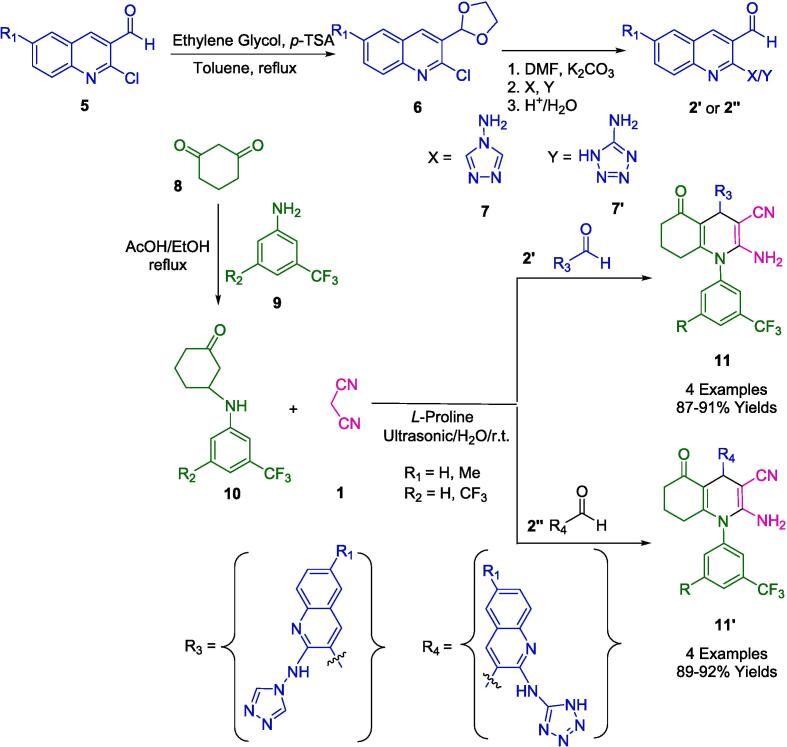
Synthesis of polyfunctionalized hexahydroquinolines.

In organic synthesis, 1,2,3-triazoles, 1,2,3-tetrazoles, and related compounds play an important role in obtaining many therapeutic compounds. The derivatives of this nucleus also have a wide spectrum of biological properties [60], [61]. Patel's group investigated the synthesis of novel biquinoline derivatives (**11′**, **11″**) through a multicomponent reaction (MCR) using 2-amino triazole/amino tetrazole-3-formyl quinoline (**2′**, **2′'**), N-enaminones (**10**) with malononitrile (**1**). In this study, the researchers employed β-enaminones (**10**) as starting materials and facilitated the reaction with ultrasound irradiation, which was catalyzed by pyridine in absolute ethanol (Scheme 2). The reaction was conducted at room temperature using an ultrasonic homogenizer, resulting in yields ranging from 87 % to 92 %. Following the procedure described in the literature, the starting material, 2-chloro-3-formyl quinoline (**5**), was prepared by the Vilsmeier-Haack reaction of acetanilide. Subsequently, it was conveniently converted into (**7**) by replacing the chlorine group at C-2 with 4-amino-1,2,4-triazole/5-amino tetrazole in the presence of anhydrous K_2_CO_3_ in DMF. The cyclohexane-1,3-dione (**8**) was reacted with substituted aniline (**9**) in ethanol with one drop of acetic acid as a catalyst to produce N-enaminones (**10**) [62].

In addition to evaluating the in *vitro* antimicrobial activity against various pathogenic strains, anti-tuberculosis (TB) activity against Mycobacterium TB H37Rv was carried out on the synthesized compounds. According to the “National Committee for Clinical Laboratory Standards”, broth microdilution was used to test the antimicrobial activity of the synthesized compounds.

**Scheme 3:**
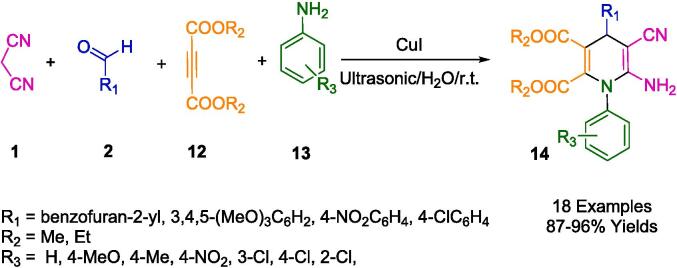
Synthesis of polysubstituted 1,4-dihydropyridines.

A series of pharmacologically interesting benzylated polysubstituted 1,4-dihydropyridines (**14**) have been synthesized by Pasha *et al.* using copper(I) iodide as a catalyst in an aqueous medium under ultrasound irradiation (Scheme 3). A synergistic effect between this catalytic system and ultrasound was demonstrated in this study. Through a one-pot cyclocondensation reaction of aromatic aldehydes (**2**), malononitrile (**1**), acetylenedicarboxylates (**12**), and arylamines (**13**) in an aqueous medium, an elegant, straightforward, and efficient method of creating 1,4-dihydropyridines (**14**) was meticulously designed. It offers several advantages over previous approaches that have been reported. These benefits include rapid response time, higher yields, more moderate conditions, convenience, environmental friendliness, and easy product isolation and purification through precipitation [63].

### 5.2 Pyridine derivatives

**Scheme 4:**
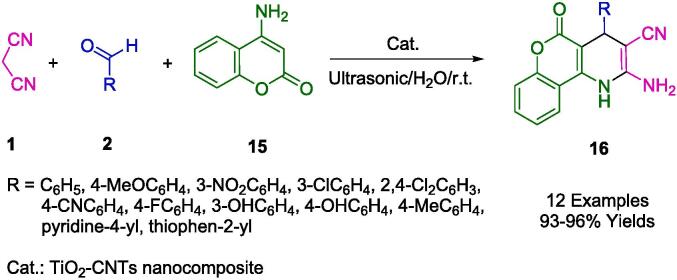
Synthesis of 2-amino-5-oxo-4-aryl-5*H*-chromeno[4,3-*b*]pyridin-3-yl cyanides.

An approach developed by Abdolmohammadi's group has been successfully applied to the synthesis of chromeno[b]pyridine derivatives (**16**) under ultrasonic irradiation in water using TiO_2_ nanoparticles immobilized on carbon nanotubes (TiO_2_-CNTs) as an efficient heterogeneous catalyst at room temperature (Scheme 4). The authors used 4-aminocoumarin (**15**), aromatic aldehydes (**2**), and malononitrile (**1**) as starting materials to synthesize a series of chromeno[*b*]pyridine derivatives using a simple and straightforward method. There was no noticeable loss of catalytic activity when the nanocomposite was reused in up to four reactions. High yields of products, short reaction times, a simple work-up procedure, and a nontoxic and reusable catalyst are some of the sustainable and economic benefits of this protocol [64].

**Scheme 5:**
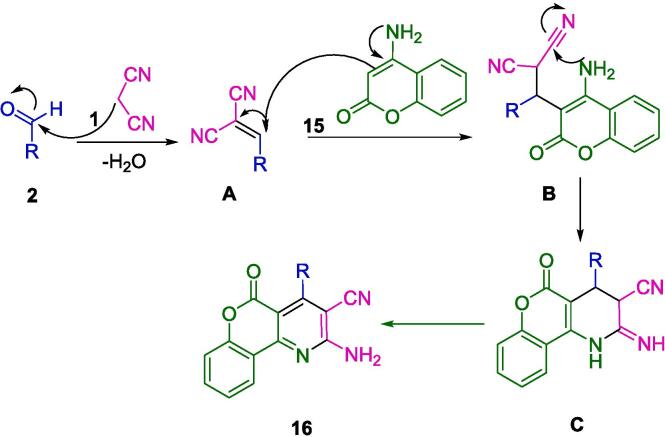
The proposed mechanism for the formation of 2-amino-5-oxo-4-aryl-5*H*-chromeno[4,3-*b*]pyridin-3-yl cyanides.

In Scheme 5, TiO_2_ nanoparticles serve as a Lewis acid-base catalyst to produce alkene **A** by reacting aromatic aldehydes (**2**) with malononitrile (**1**). Additionally, TiO_2_ nanoparticles are employed as a catalyst for the Michael addition of 4-aminocoumarin (**15**) to alkene **A**, resulting in the formation of Michael adduct **B**. The intermediate **C** undergoes tautomerization and aromatization, followed by the cycloaddition of amino groups to the cyano moiety, ultimately yielding the product (**16**).

**Scheme 6:**
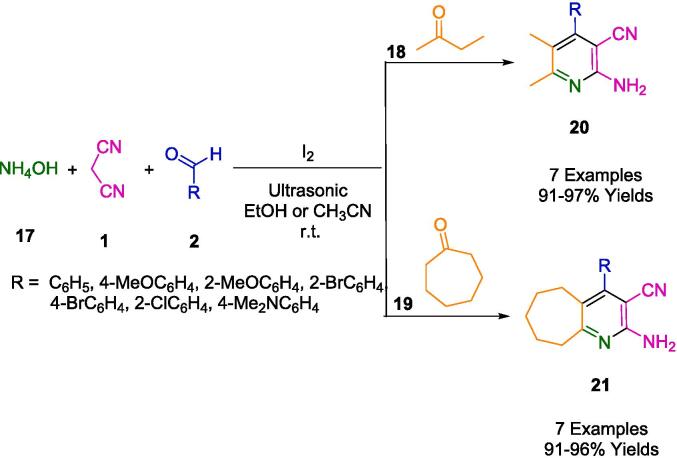
Synthesis of polyfunctionalized pyridine-annulated heterocyclic compounds.

By condensing aldehyde (**2**), malononitrile (**1**), aliphatic/cyclic ketones (**18**, **19**), and ammonium hydroxide (**17**) in acetonitrile or ethanol under ultrasound irradiation at room temperature (r.t.), Paladala *et al.* demonstrated iodine-promoted facile cost-effective procedures for the production of pyridoimidazoisoquinolines (**20**,**21**) at room temperature (Scheme 6). Under ultrasonic irradiation, a catalytic amount of I_2_ in EtOH catalyzed the reaction. In this way, 91–97 % of the product was produced. Their in-silico studies present an exceptional binding affinity to interactions with hydrogen as well as the proposed ultrasound-assisted eco-friendly, cost-effective protocol [65].

**Scheme 7:**
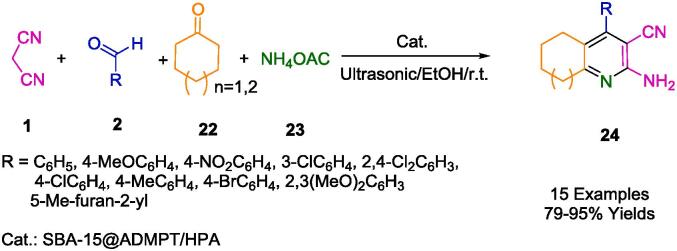
Synthesis of 2-amino-3-cyanopyridine derivatives.

A nanohybrid catalyst, denoted as SBA-15@Triazine/H_5_PW_10_V_2_O_40_, was meticulously engineered by Ghanbari *et al.*, with a specific emphasis on environmentally benign synthetic methodologies facilitated by reusable heterogeneous nanocatalysts. An ADMPT linker was covalently affixed to the SBA-15 mesoporous silica framework, serving as the scaffold for the chemical anchoring of Keggin-type heteropolyacid, H_5_PW_10_V_2_O_40_, onto the surface. Utilizing these nanohybrids as a sustainable, efficacious, and highly recyclable catalytic system, the synthesis of 2-amino-3-cyanopyridin derivatives (24) was accomplished via a one-pot multicomponent condensation of aldehyde (2), malononitrile (1), cyclic ketones (22), and ammonium acetate (23) under the influence of ultrasound waves (as outlined in Scheme 7). The resultant yields were commendable, ranging from good to excellent (79–95 %), and the reaction duration was comparatively brief. Notably, the nanocatalysts were recoverable and were reutilized in at least five successive reactions after appropriate drying and reactivation, without incurring any substantial loss in product yield [66].

**Scheme 8:**
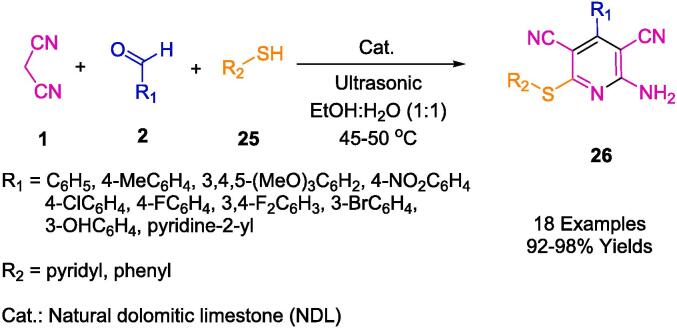
Synthesis of 2-amino-4-(hetero)aryl-3,5-dicarbonitrile-6-sulfanylpyridines.

Natural Dolomitic Limestone (NDL) constitutes an ecologically responsible, cost-effective, and inherently non-toxic amalgamation of calcium and magnesium carbonate, offering particular utility in the realm of heterogeneous catalysis. Developed by Nallagondu *et al.*, this heterogeneous catalyst (as delineated in Scheme 8) has proven efficacious in the synthesis of highly functionalized pyridine (**26**) through a reaction medium of ethanol and water. Conducted at a temperature range of 45–50 °C, this multicomponent reaction (MCR) protocol rendered the desired products in elevated yields. The methodology accommodates a wide range of substrates, proffers a streamlined reaction profile, and necessitates minimal reaction time, all while delivering excellent isolated yields. Remarkably, chromatographic purification of the products is rendered superfluous, and the catalyst boasts a reusability quotient of up to seven cycles. In a comparative analysis with extant catalysts, this catalyst emerges as a more environmentally congruent alternative for the synthesis of *N*-heterocycles [67].

### 5.3 Pyrimidine

**Scheme 9:**
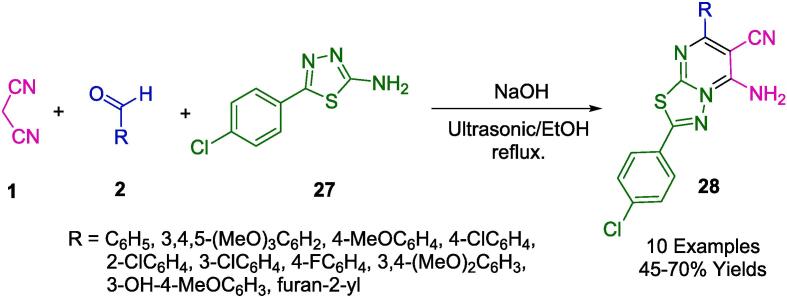
Synthesis of thiadiazolo(3,2-*α*)pyrimidine-6-carbonitrile derivatives.

Recognized for their prominence in the field of medicinal chemistry, pyrimidine and its various derivatives hold the status of central heterocyclic compounds [68], [69], [70]. In alignment with this significance, Nikalje *et al.* have designed an eco-conscious, expeditious, and facile one-pot synthesis of thiadiazolo(3,2-*α*)pyrimidine-6-carbonitrile derivatives (**28**), employing ultrasound irradiation to achieve yields ranging from reasonable to good (as described in Scheme 9). Complementing these advancements, our research endeavors focus on formulating environmentally sustainable methodologies for acquiring these critical heterocyclic frameworks. Specifically, we have pioneered a one-pot condensation approach that utilizes ultrasonic irradiation, facilitated by NaOH under reflux conditions and employs ethanol as the eco-compatible solvent. This method integrates 5-(4-chlorophenyl)-1,3,4-thiadiazol-2 amine (**27**), aromatic aldehydes (**2**), and malononitrile (**1**) to achieve the desired synthesis.

In *vitro* tests were conducted on four human tumor cell lines with these compounds. According to the GI50 values of HeLa, K562, PC-3, and MCF-7 cancer cells, compound 4i, which has substituted 3-hydroxy-4-methoxyphenyl, has the highest values of 32.7 m, 55.3 m, 34.3 m, and 28.9 m compared with all synthetic derivatives. The thrymidylate synthase enzyme showed good binding modes to the newly synthesized compounds. The analyzed compounds also exhibited drug-like properties based on their ADME properties [71].

**Scheme 10:**
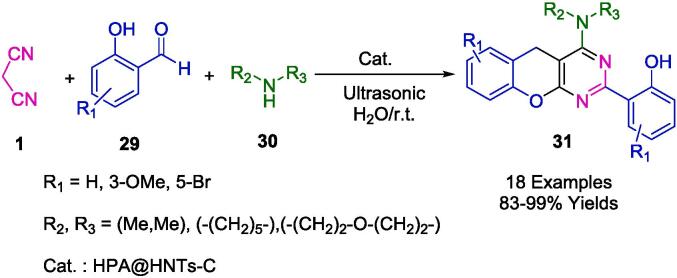
Synthesis of benzopyranopyrimidines.

Through the incorporation of heteropolyacids into creatin-functionalized halloysite clay, the Sadjadi group developed an efficient heterogeneous catalyst that promotes organic transformation via HPA heterogenization via its immobilization on creatin-functionalized HNTs. As a catalyst, HPA@HNTs-C was studied and investigated for the synthesis of benzopyranopyrimidines (**31**) via MCR with 2-hydroxybenzaldehydes (**29**), malononitrile (**1**), and amines (**30**) (Scheme 10). Using water as a green solvent, the reaction was conducted under ultrasound irradiation at room temperature with yields ranging from 83 % to 99 %. It is also possible to prevent heteropolyacid leakage by immobilizing it on a halloysite that has been functionalized with creatin. Catalysts used in this protocol were tested for their reusability and were found to be able to be reused up to five times. In terms of yield, reaction time, and eco-friendliness, the protocol proved to be efficient [72].

**Scheme 11:**
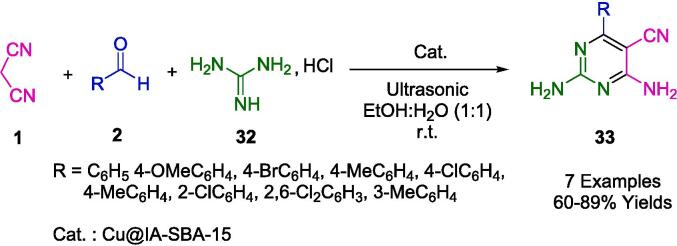
Synthesis of 5-pyrimidinecarbonitriles.

By developing a one-pot process for synthesizing pyrimidine derivatives (**33**) using malononitrile (**1**), guanidine hydrochloride (**32**), and different aromatic aldehydes (**2**) under ultrasound irradiation in EtOH:H_2_O (1:1) as a green solvent at room temperature (Scheme 11). The Safaei Ghomi group was motivated to search for greener synthetic routes to obtain heterocycles. An isatoic anhydride anchor was anchored onto amino-functionalized SBA-15 under ultrasonic irradiation to produce a heterogeneous hybrid catalyst, Cu@IA-SBA-15. An ordered 2D-hexagonally ordered mesoporous bidentate ligand was synthesized by grafting Cu (II) to capture the desired product. In addition to its impressive performance and environmental friendliness, it produces high yields and short reaction times. After the recovery process, the nanocomposite was successfully utilized in four consecutive reaction cycles, with minimal reduction in catalytic activity [73].

**Scheme 12:**
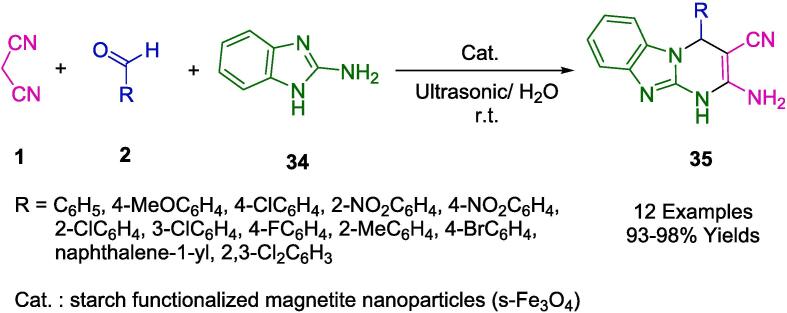
Synthesis of imidazopyrimidines.

The Srivastava research group has synthesized a cohort of biologically salient imidazopyrimidine derivatives (**35**) via an environmentally benign one-pot multicomponent methodology. This process involves the condensation of malononitrile (**1**), 2-aminobenzimidazole (**34**), and aromatic aldehydes (**2**), facilitated by starch-functionalized magnetite nanoparticles (s-Fe_3_O_4_) serving as non-toxic, versatile biocatalysts. Notably, the reactions transpire in an aqueous medium at ambient temperature, aided by ultrasonic irradiation (as outlined in Scheme 12). This nanocomposite catalyst exhibits robust reusability, maintaining its catalytic efficiency over six iterative cycles. The method offers numerous advantages, including facile product isolation, exceptional atom economy, and mild operational conditions. Furthermore, it obviates the need for column chromatography and incorporates the added benefit of magnetic separability for catalyst recovery [74].

**Scheme 13:**
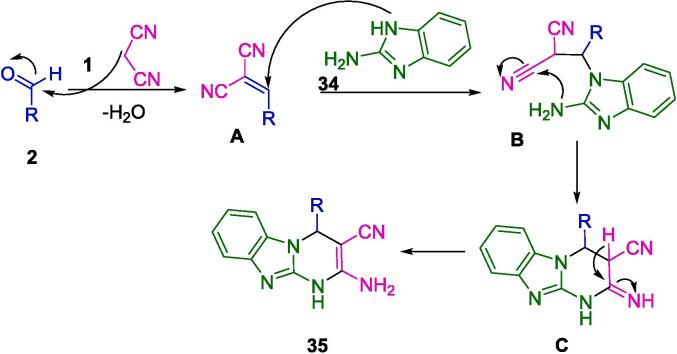
Plausible mechanism for s-Fe_3_O_4_ catalyzed synthesis of imidazopyrimidine.

In Scheme 13, a mechanism is presented for the synthesis of imidazopyridine derivatives (**35**), catalyzed by starch-functionalized magnetite nanoparticles (s-Fe_3_O_4_). The catalyst serves to polarize the carbonyl group in aromatic aldehydes (**2**), thereby enhancing its electrophilic nature. This facilitates the condensation with malononitrile (**1**), leading to the formation of arylidene malononitrile intermediates (**A**). The final product (**35**) is achieved through a Michael addition of 2-amino benzimidazole (**34**) to the arylidenenitrile intermediate (**A**), followed by an intermolecular cyclization event (**B**).

**Scheme 14:**
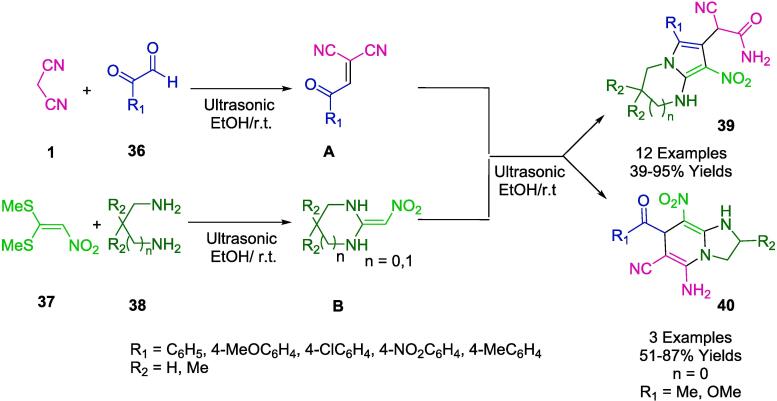
Synthesis of imidazo[1,2-a]pyridine-6-carbonitrile derivatives.

Alizadeh group presented an environmentally friendly, catalyst-free, and ultrasound-assisted tandem pseudo-four-component reaction synthetic approach. In this process, phenyglyoxal derivatives (**36**), malononitrile (**1**), 1,1-bis(methylsulfanyl)-2-nitroethene (**37**), and diamines (**38**), under mild conditions were condensed for the synthesis of pyrrolo[1,2-*a*]pyrimidin-7-yl)acetamides (**39**), and 1*H*-pyrrolo[1,2-*a*]imidazol-6-yl)acetamides (**40**) in EtOH as a green solvent to reach a product with reasonable to excellent yields (39–95 %) (Scheme 14). The structure of 2-cyano-2-(8-nitro-6-phenyl-1,2,3,4-tetrahydropyrrolo[1,2-*a*]pyrimidin-7-yl)acetamide was characterized and verified by X-ray analysis. This efficient and green protocol has remarkable advantages, such as abundant starting materials, simple operation, clean reaction profile, easy purification without traditional methods (column chromatography and crystallization), excellent tolerance to various substituents, and relatively short reaction time [75].

**Scheme 15:**
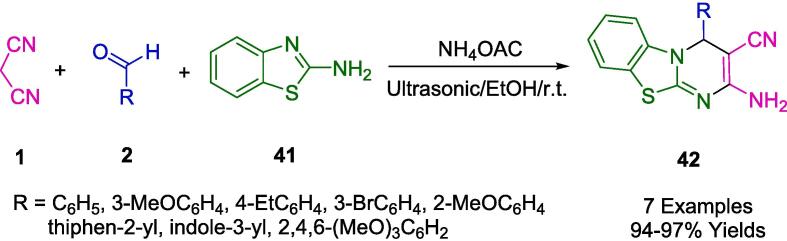
Synthesis of benzo[4], [5]thiazolo[3,2-a]pyrimidine derivatives.

In a study conducted by Jonnalagadda, an optimized one-pot synthesis was developed for the generation of novel benzo[4], [5]thiazolo[3,2-*a*]pyrimidine scaffolds (**42**). This procedure entailed the amalgamation of 2-aminobenzothiazole (**41**), various aldehydes (**2**), and malononitrile (**1**) (Scheme 15). Utilizing ammonium acetate as a catalyst and ethanol as a green solvent, the reaction proceeded at ambient temperature under the influence of ultrasonic irradiation. Notably, the protocol achieved remarkable yields ranging from 94 to 97 %. Additional merits of this methodology include the elimination of the need for column chromatography, the utilization of an environmentally benign solvent, and the accommodation of varied functional groups—all while operating under mild conditions at room temperature [76].

**Scheme 16:**
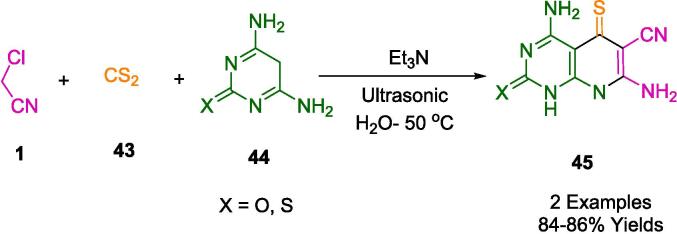
Synthesis of 5-thioxopyridopyrimidine derivatives.

Based on the results of Ali *et al.*, a facile green one-pot and three-component reaction has been developed to synthesize a series of novel 5-thioxopyridopyrimidine derivatives (**45**) with readily available starting materials carbon disulfide (**43**), malononitrile (**1**), and pyrimidine compounds (**44**) catalyzed by triethylamine under ultrasonication in the presence of water as a solvent at 50 °C (Scheme 16). Besides its simplicity, this method is environmentally friendly, produces high yields, and takes only a short amount of time. This method provides inexpensive catalysts, rapid reactions, excellent yields, green solvent, ultrasound irradiation, and avoids column chromatography and hazardous solvents [77].

## 6 O-Heterocyclic compounds

### 6.1 Pyran derivatives

**Scheme 17:**
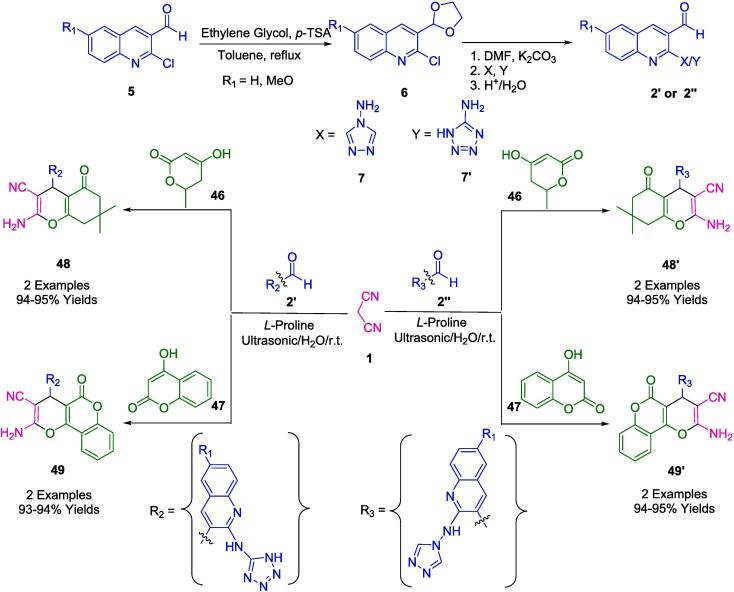
Synthesis of pyrano-chromene and benzopyrano-chromene derivatives.

In the work presented by Patel *et al.*, a series of pyranochromene and benzopyranochromene derivatives (**48**, **49**, **48′**, **49′**) were efficiently synthesized via a one-pot multicomponent reaction (MCR). This green synthetic route employed *L*-proline in conjunction with specialized synthetic aldehydes 6-(un)substituted-2-((4*H*-1,2,4-triazol-4-yl)amino)-quinoline-3-carbaldehyde (**2′**) or 6-(un)substituted-2-((1*H*-tetrazol-5-yl)amino)-quinoline-3-carbaldehyde (**2′'**) to catalyze the condensation with either 4-hydroxycoumarin (**47**) or 4-hydroxy-6-methyl pyran (**46**), and malononitrile (**1**) (Scheme 17). The use of water as a solvent and the application of ultrasound irradiation to initiate the reactions are hallmarks of this eco-friendly protocol. The approach is noted for its mild reaction conditions, high atom efficiency, rapid completion time, and exceptionally high yields, making it a superior option when compared to other methods [78].

**Scheme 18:**
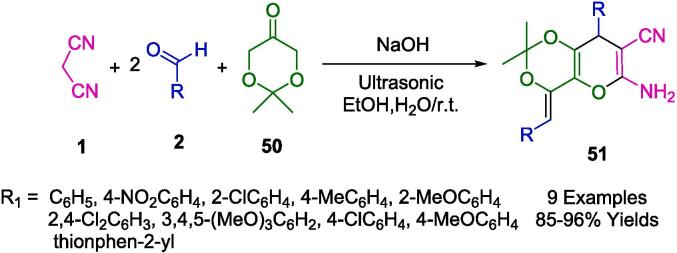
Synthesis of a series of dihydropyrano[3,2-d][1], [3]dioxin heterocycles.

Mojtahedi *et. al* reported an ultrasound-based one-pot cyclocondensation of 1,3-dioxane-5-one (**50**) with malononitrile (**1**) and two equivalents of aromatic aldehydes (**2**)*.* The process of ultrasonic irradiation of aqueous sodium hydroxide produces pyrano[3,2-*d*][1], [3]dioxin derivatives (**51**) in high yield (Scheme 18) [79].

**Scheme 19:**
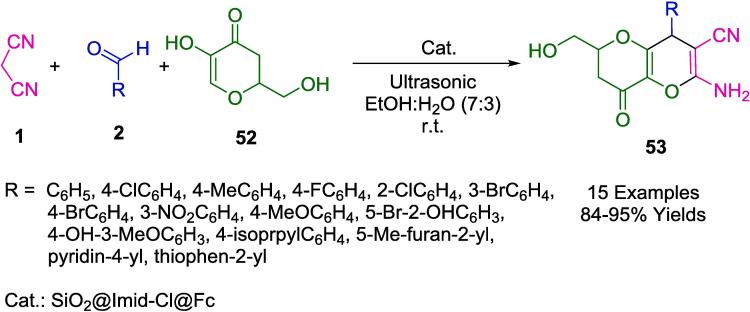
Synthesis of pyrano[3,2-*b*]pyrans.

Teimuri-Mofrad *et al.* have devised an eco-conscious synthetic route employing silica nanosphere-supported ionic liquids functionalized with ferrocene (SiO_2_@Imid-Cl@Fc) as a catalyst (Scheme 19). This catalytic system facilitates the formation of pyrano[3,2-*b*]pyran derivatives (**53**) via a one-pot, three-component reaction involving malononitrile (**1**), kojic acid (**52**), and a selection of aromatic aldehydes (**2**). The reaction milieu -an aqueous ethanol solution- and the ambient temperature conditions under sonication not only optimize the reaction kinetics but also adhere to the tenets of green chemistry. The reusability of the catalyst, without significant degradation of its efficacy for up to five consecutive cycles, underscores its potential for application in industrial processes, promoting sustainability and economic viability. This innovation epitomizes a strategic shift towards more sustainable organic synthesis, prioritizing environmental compatibility without compromising the synthetic efficiency [80].

**Scheme 20:**
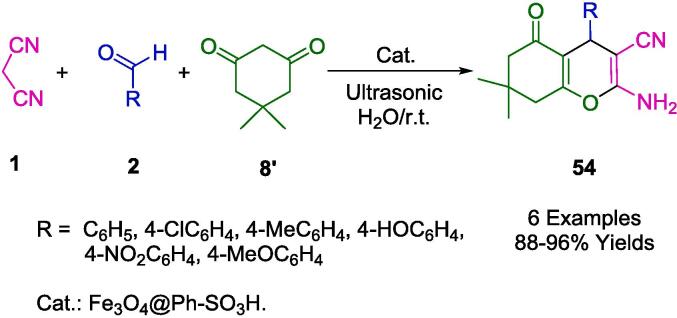
Synthesis of tetrahydrobenzo[*b*]pyran derivatives.

In an aqueous medium and under ambient conditions, Elhamifar *et al.* synthesized a suite of tetrahydrobenzo[*b*]pyrans (**54**) leveraging a robust nanocatalyst Fe_3_O_4_@Ph-SO_3_H, composed of magnetic iron oxide with a phenylsulfonic acid functionalized shell (Scheme 20). This catalytic system was thoroughly characterized and evidenced exceptional efficacy in the aforementioned synthetic application, with the aid of ultrasonic agitation. The synthetic process for the catalyst entailed modifying the magnetic iron oxide core with 1,4-bis(triethoxysilyl)benzene (BTEB), followed by sulfonation of the aromatic extensions. The prepared nanocatalyst demonstrated remarkable resilience, maintaining its catalytic prowess for no fewer than nine successive runs, which is indicative of its stability and sustainable utility. The research articulated by Elhamifar *et al.* underscores the profound advantages of the employed methodology, such as the brisk reaction kinetics, truncated reaction duration, uncomplicated separation of product from catalyst, minimized energy expenditure by operating at room temperature, and the superior yields of the desired products. This approach encapsulates the quintessence of green chemistry, reinforcing the drive towards eco-friendlier synthetic strategies in chemical processes [81].

**Scheme 21:**
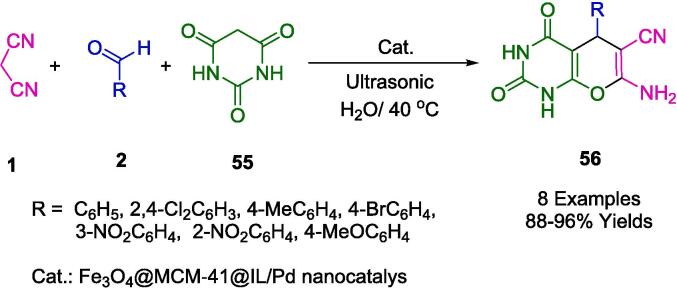
Synthesis of pyrano[2,3‐*d*]pyrimidine derivatives.

The authors' inventive methodology involved the development of a composite heterogeneous nanocatalyst, labeled Fe_3_O_4_@MCM@IL/Pd, through a post-grafting technique that incorporates a magnetic core with a shell functionalized with an ionic liquid and palladium. This catalyst was adeptly utilized for the synthesis of pyrano[2,3-*d*]pyrimidine derivatives (**56**), compounds known for their range of biological activities (Scheme 21). The synthesis process itself was carried out in an aqueous medium under the influence of ultrasonic waves at a moderate temperature of 40 °C, resulting in notably high to excellent yields and underscoring the efficiency of the catalytic system. Exhibiting exceptional reusability, the catalyst could be magnetically separated and employed for up to 11 cycles with negligible loss in activity or selectivity, thus demonstrating remarkable endurance. The quick reaction turnover, combined with the catalyst's stability and the straightforward separation from the reaction products, makes it an exemplary tool in the realm of green chemistry. The catalyst’s performance is indicative of its potential to enable more sustainable chemical processes, operating effectively at moderate temperatures and obviating the need for harsh organic solvents [82].

**Scheme 22:**
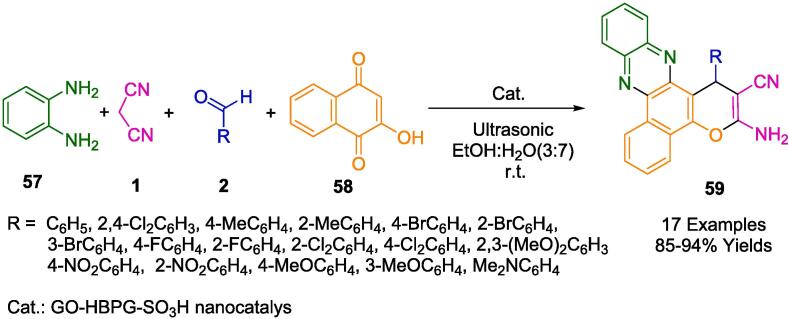
Synthesis of benzo[*a*]pyrano-[2,3-*c*]phenazine compounds.

The pioneering approach by Naeimi *et al.* encapsulates the principles of green chemistry, leveraging a nanocatalyst enhanced by the unique properties of graphene oxide to facilitate the synthesis of benzo[*a*]pyrano[2,3-*c*]phenazines (**59**) (Scheme 22). The process converges on a one-pot, sonochemical method that operates at ambient temperatures, employing a mixture of ethanol and water as the solvent medium to combine 2-hydroxynaphthalene-1,4-dione (**58**), *o*-phenylenediamine (**57**), various aldehydes (**2**), and malononitrile (**1**). The nanocatalyst itself is a testament to advanced material design, where hyperbranched polyglycerol (HBPG) is grafted onto graphene oxide and subsequently functionalized with sulfonic acid groups. This results in a high density of acidic functional groups on the catalyst’s surface, which is pivotal for the reaction's efficiency. A salient feature of this methodology is the sustainable aspect of the catalyst's life cycle, it can be easily recovered and recycled for at least five subsequent runs without a significant decrease in performance. The sonochemical conditions that underpin this synthetic route provide several other benefits, including the rapidity of the reaction, high product yields, and the avoidance of more environmentally detrimental conditions typically associated with chemical syntheses [83].

**Scheme 23:**
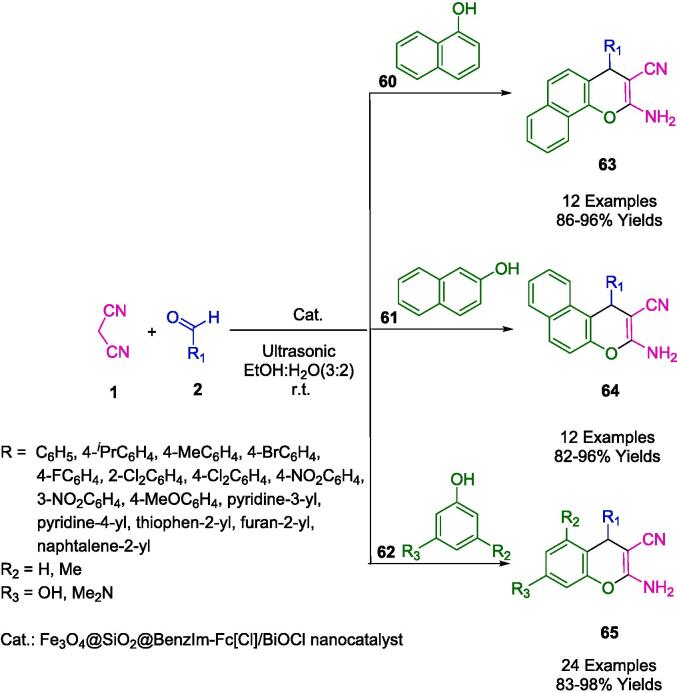
Ultrasound-assisted synthesis of diverse 2-amino-4*H*-chromenes.

An efficient and reusable nanocatalyst was developed by Mohammadi *et al.* by using a simple chemical co-precipitation method for Fe_3_O_4_@SiO_2_ – BenzIm-Fc[Cl]/BiOCl nano-composite (Scheme 23). It was the first time that an ionic liquid, ferrocene, and BiOCl are combined to form a magnetic nano-catalyst. In the presence of EtOH:H_2_O (3:2) as a solvent, and ultrasonic irradiation at room temperature, the nanocomposite was evaluated for one-pot synthesis of a wide variety of 2-amino-3-cyano-4*H*-chromene derivatives (**63**, **64**, **65**). An ultrasound-assisted method was developed for producing 4*H*-chromene derivatives from aldehydes (**2**), malononitrile (**1**), and active phenolic compounds 2-naphthol (**61**), 1-naphthol (**60**), substituted phenol (**62**) at room temperature using a one-pot, three-component reaction. A number of advantages can be observed in the sonosynthesis protocol studied in this article. There are many advantages inherent in this process, such as short reaction times, operational simplicity, green reaction conditions, high yields, and ease of work-up and purification. In terms of recovery and reusability, the nanocomposite was excellent. Recovering the material was as easy as washing it with EtOH and drying it. It was possible to reuse this nanocomposite up to six times while maintaining its catalytic activity [84].

**Scheme 24:**
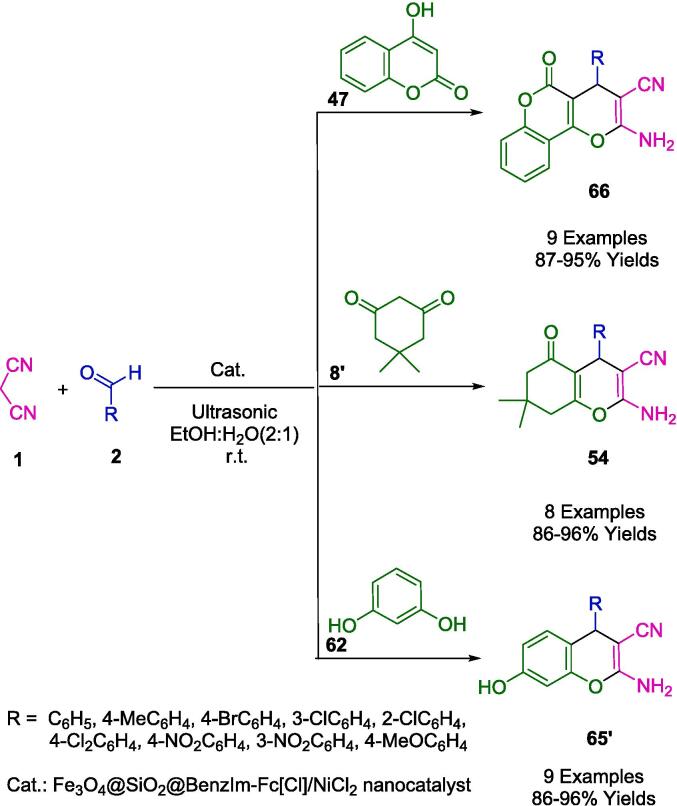
Synthesis of a wide variety of pyran derivatives.

The synthesis of many types of pyran derivatives (**67**, **54**, **65′**) in ethanol-water (2:1) under ultrasound irradiation has been reported using versatile and highly efficient heterogeneous catalysts (Scheme 24). A simple chemical coprecipitation method was also used to synthesize a magnetically recoverable Fe_3_O_4_@SiO_2_-BenzIm-Fc[Cl]/NiCl_2_ nanoparticle. This section discusses synthetic protocols involving aromatic aldehydes (**2**) in a one-pot reaction with malononitrile (**1**), 4-hydroxycoumarin (**47**), dimedone (**8′**), or resorcinol (**62**), as well as Fe_3_O_4_@SiO_2_@BenzIm-Fc[Cl]/NiCl_2_ nanoparticles, developed by Mohammadi *et al.* In addition to its simplicity, high yields, and ease of use, the proposed catalytic method exhibits some notable advantages. A magnet was used to separate the new magnetic nanoparticles and re-use them six times without significantly affecting their catalytic activity [85].

**Scheme 25:**
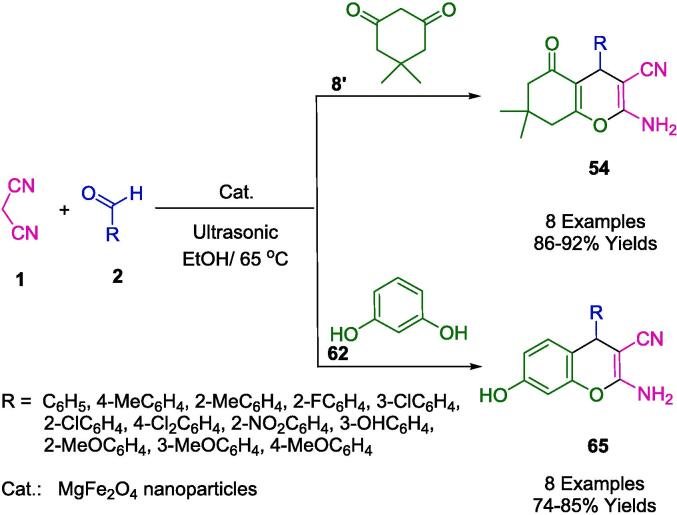
Synthesis of tetrahydrobenzo[*b*]pyrans and 2-amino-7-hydroxy-4*H*-chromenes.

Rouhani *et al.* successfully synthesized a series of 2-amino-7-hydroxy-4*H*-chromene and tetrahydrobenzo[*b*]pyran compounds (**54**, **65**) utilizing magnesium ferrite (MgFe_2_O_4_) nanoparticles as an effective heterogeneous catalyst. This synthesis was achieved through the application of ultrasound irradiation at a temperature of 65 °C in an ethanol medium (as illustrated in Scheme 25). The chemical process involves a reaction among various aldehydes (**2**), malononitrile (**1**), and resorcinol (**62**) or dimedone (**8′**), facilitated by the presence of the MgFe_2_O_4_ nanoparticulate catalyst under sonication. The MgFe_2_O_4_ nanoparticles demonstrated the additional advantage of magnetic recoverability, allowing for their facile separation with an external magnet and the possibility of being recycled up to four times with negligible diminution in their catalytic efficacy. This methodology is underscored by its straightforwardness, high efficiency, environmental compatibility, and expedited purification process [86].

**Scheme 26:**
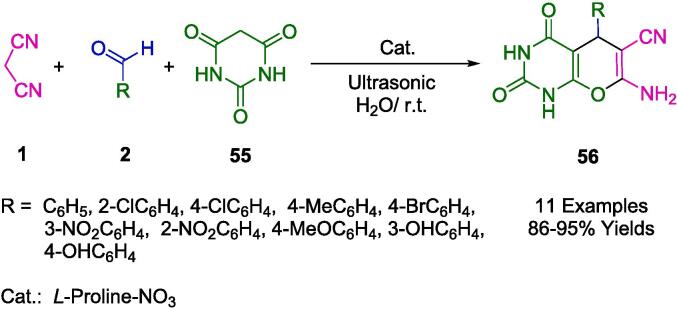
Synthesis of pyrano[2,3-*d*] pyrimidine diones derivatives.

The research team led by More has elucidated the catalytic efficiency of *L*-Proline nitrate, showcasing its efficacy as a homogenous and environmentally benign catalyst. Employing this catalyst, the group adeptly synthesized pyrano[2,3-*d*]pyrimidine diones (**56**) at ambient conditions, leveraging ultrasonic irradiation as depicted in Scheme 26. The formation of *L*-Proline nitrate, derived from a simple combination of sodium nitrate with *L*-proline, represents an ecologically conscious catalytic system. This catalyst exhibited exceptional catalytic activity when employed in a solvent-free, ultrasound-assisted multicomponent reaction (MCR) involving substituted aromatic aldehydes (**2**), malononitrile (**1**), and 1,3-dimethyl barbituric acid (**55**), facilitating the synthesis of the pyrimidine diones at room temperature [87].

Ionic liquids formulated from *L*-Proline-NO_3_ represent a subclass of amino acid ionic liquids (AAILs) that are not only easily derivable from naturally occurring substrates but also notable for their sustainability credentials. The facile synthesis of *L*-Proline nitrate ionic liquids (ILs) from readily accessible commercial sources underscores their alignment with green chemistry principles, given their biocompatibility and non-toxic nature. These organocatalytic ILs display a noteworthy potential for reusability, maintaining their efficacy for up to six consecutive uses without substantial diminution in yield. The protocol associated with these ILs also highlights an eco-conscious approach by enabling solvent recovery and reuse. Furthermore, the operational simplicity of this method, coupled with its rapid reaction kinetics and the circumvention of column chromatography for product purification, solidifies its environmental soundness. This process, which leverages aqueous media as a reaction solvent, emphasizes sustainability and can efficaciously be employed for at least five successive synthetic cycles.

**Scheme 27:**
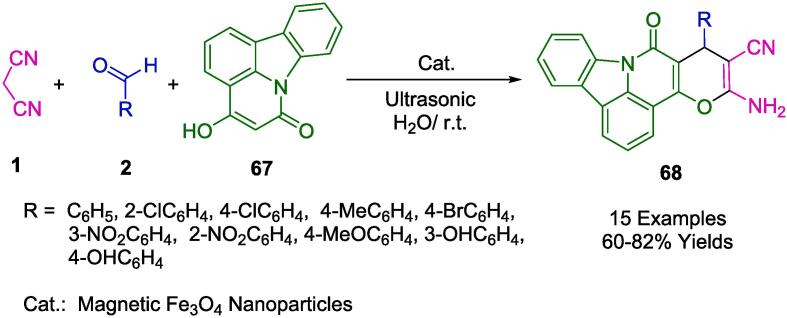
Synthesis of 5-amino-8-oxo-7-phenyl-5,6,7,8 dihydropyrano[2′,3′,4,5]pyrido[3,2,1-*jk*]carbazol-6-carbonitriles.

In their innovative approach to the synthesis of a new array of pyrano-pyrido-carbazol derivatives (PPCDs) (**68**), Verma *et al.* employed the environmentally conscious technique of ultrasound-facilitated multicomponent reactions (MCR). For this process, depicted in Scheme 27, Fe_3_O_4_ magnetite nanoparticles (Fe_3_O_4_ MNPs) were introduced as a novel heterogeneous catalyst. The synthesis was conducted in an aqueous medium, combining 4-hydroxypyridocarbazolone (**67**), aryl/heteroaryl aldehyde (**2**), and malononitrile (**1**), all under ambient temperature and subjected to ultrasonication. Post-reaction, the Fe_3_O_4_ MNPs were efficiently separated from the reaction mixture via an external magnet and demonstrated remarkable stability, being recyclable for up to seven successive uses with minimal loss in activity. The ultrasonic energy not only expedited the PPCD synthesis but also ensured the uniform dispersion of the MNPs within the aqueous medium. The advantages of this protocol are manifold, highlighting the expedient assembly of novel PPCDs, the cost-effectiveness of the catalyst, adherence to ecological safety standards, and a strong alignment with the protocols of green chemistry [88].

**Scheme 28:**
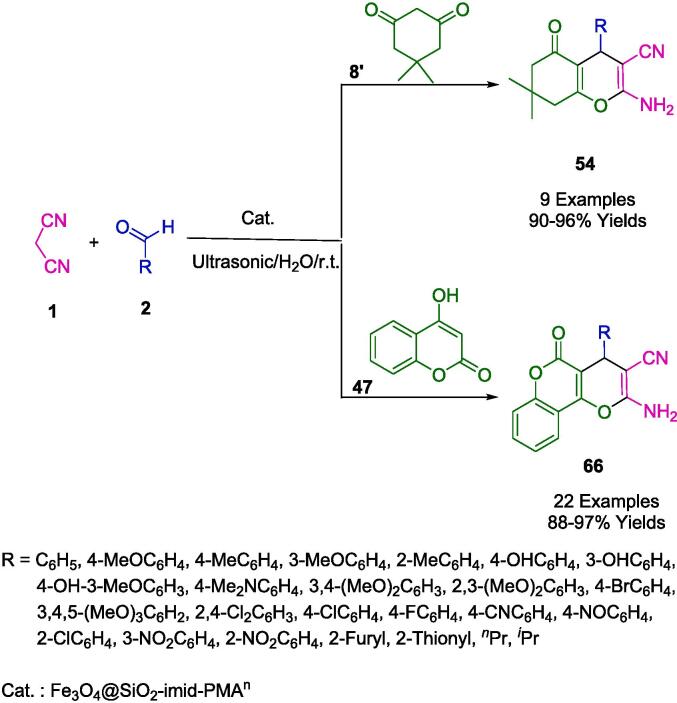
Synthesis of tetrahydro-4*H*-benzo[*b*]pyrans and dihydropyrano[3,2-*c*]chromenes.

Esmaeilpour *et al.* have articulated the design of a sustainable synthetic strategy utilizing a Fe_3_O_4_@SiO_2_-imid-PMAn-based nanocatalyst, which serves both as a magnetic and recyclable catalyst within the context of synthesizing tetrahydrobenzo[*b*]pyran (**54**) and 3,4-dihydropyrano[*c*]chromene derivatives (**66**), as delineated in Scheme 28. This one-pot, three-component reaction, effectuated in an aqueous medium, engages malononitrile (**1**), a variety of aldehydes (**2**), and dimedone (**8′**) or 4-hydroxycoumarin (**47**), proceeding at room temperature. The methodology introduced is operationally uncomplicated, engenders high yields, requires minimal reaction time, and obviates the need for elaborate work-up or purification processes. The catalyst in question not only excels in its catalytic role in water but is also facile to prepare, eschews the use of deleterious organic solvents, and exhibits significant thermal stability. Furthermore, it can be seamlessly separated from the reaction milieu owing to its magnetic properties, thereby enhancing its utility as an efficient heterogeneous catalyst in comparison to its contemporaries. The magnetically retrievable nature of the catalyst bestows upon it an economic and environmental edge, with the catalyst retaining its activity over eight successive reaction cycles without marked degradation [89].

**Scheme 29:**
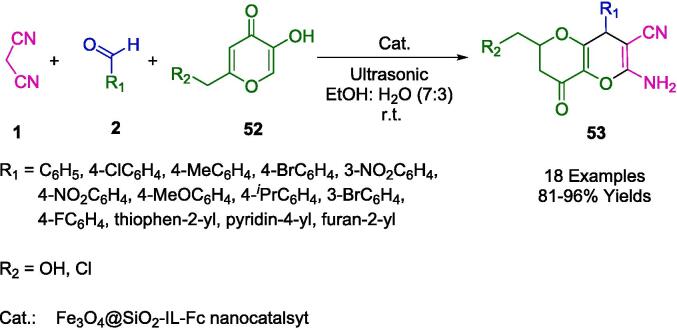
Synthesis of pyrano[3,2-*b*]pyran derivatives.

Teimuri-Mofrad *et al.* innovated a novel magnetic nanocatalyst, nano Fe_3_O_4_@SiO_2_-IL-Fc, which incorporates a ferrocene-containing ionic liquid supported on magnetically coated silica nanoparticles. This advanced heterogeneous nanocatalyst facilitates the ultrasound-assisted synthesis of pyrano[3,2-*b*]pyran compounds (**53**) via a one-pot method, as delineated in Scheme 29. The procedure involves a three-component condensation of malononitrile (**1**), various aldehydes (**2**), and kojic acid derivatives (**52**) under ultrasonic irradiation at ambient temperature. This technique was also compared to conventional stirring methods, with the ultrasonically driven reactions displaying advantages such as accelerated reaction kinetics, streamlined operational setup, and enhanced yields, coupled with simpler purification protocols due to shortened reaction durations. Moreover, the nanocatalyst can be efficiently separated from the reaction system with a magnet and boasts impressive reusability for at least six consecutive cycles without a significant reduction in catalytic performance [90].

**Scheme 30:**
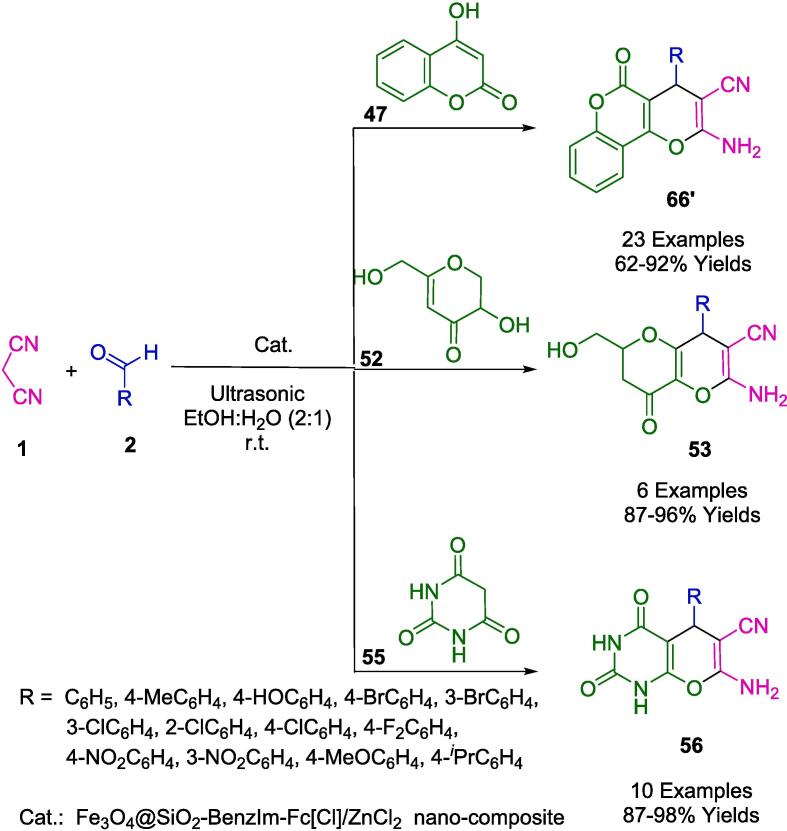
Synthesis of pyrano[2,3-*d*]pyrimidine derivatives, pyrano[3,2-*b*]pyran derivatives, and chromeno[2,3-*d*]pyrimidine derivatives.

Safa and colleagues have outlined an efficient synthesis route for the creation of magnetically separable Fe_3_O_4_@SiO_2_-BenzIm-Fc[Cl]/ZnCl_2_ nanoparticles, as presented in Scheme 30. Utilizing an uncomplicated, straightforward, and highly efficacious sonication-assisted multicomponent reaction methodology, they succeeded in the synthesis of chromeno[2,3-*d*]pyrimidine derivatives (**66′**), pyrano[3,2-*b*]pyran derivatives (**53**), and an array of pyrano[2,3-*d*]pyrimidine derivatives (**56**). The innovative nanocatalyst Fe_3_O_4_@SiO_2_-BenzIm-Fc[Cl]/ZnCl_2_ was engaged in the condensation process involving aldehydes (**2**), malononitrile (**1**), 4-hydroxycoumarin (**47**), kojic acid (**52**), and barbituric acid (**55**). It was established that this mesoporous catalyst retains its efficacy over six cycles of reuse. The catalytic approach introduced is characterized by numerous benefits, including high product yields, ease of work-up and purification, abbreviated reaction durations, operational ease, and adherence to green chemistry principles [91].

**Scheme 31:**
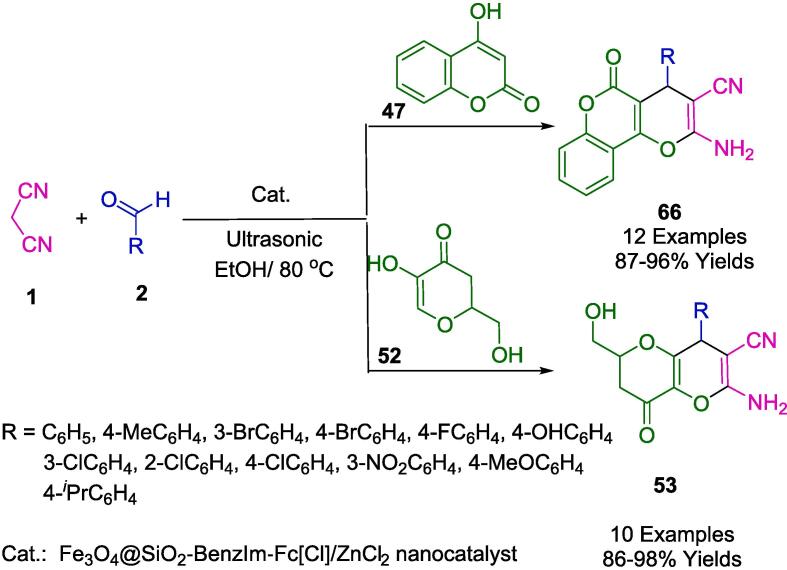
Synthesis of pyrano[3,2-*c*]chromenes and tetrahydropyrano[3,2-*b*]pyrans.

In their exploration of eco-friendly heterogeneous catalysis, Ghashang *et al.* focused on the ultrasonic facilitation of synthesizing pyran derivatives (**66**, **53**). They investigated the utilization of green solvents alongside a blend of malononitrile (**1**), activated enols (**47**, **52**), and aldehydes (**2**) as condensing agents. Their research, outlined in Scheme 31, delved into the employment of Zn_2_SnO_4_-SnO_2_ nano-composite as a potent and environmentally benign heterogeneous catalyst, capable of effectively generating the desired pyran nuclei at a controlled temperature of 80 °C. The catalyst itself was synthesized via a sol–gel process in a medium derived from sour orange water. The ultrasonic method championed by the researchers is notable for securing high yields and diminishing reaction times while requiring only minimal quantities of nanocatalyst under gentle conditions and a straightforward experimental setup. The protocol also allows for the catalyst to be efficiently separated from the reaction mixture and reused in subsequent syntheses [92].

**Scheme 32:**
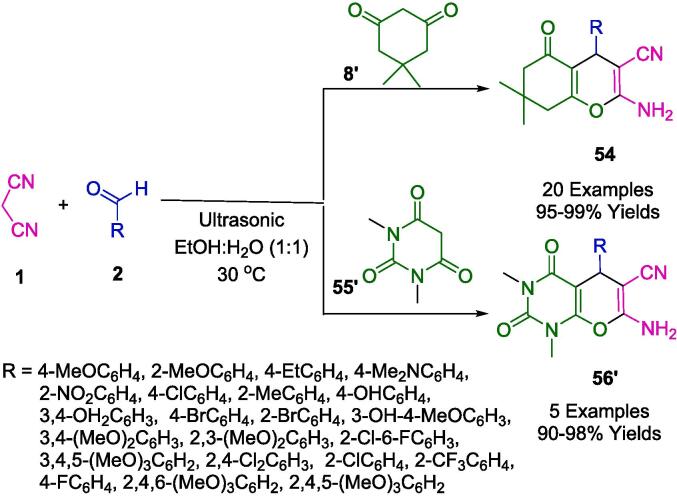
Synthesis of polyfunctionalized pyran derivatives.

Janagadda's team developed an environmentally favorable and highly effective catalyst-free synthetic procedure for the fabrication of two categories of polyfunctionalized pyran derivatives (**54**, **56′**). This novel approach involves a one-pot reaction that unites aromatic aldehydes (**2**), malononitrile (**1**), and either dimedone (**8′**) or 1,3-dimethyl barbituric acid (**55′**), employing a mixture of ethanol and water (EtOH: H_2_O, 1:1 v/v) as the reaction solvent. The process is facilitated by ultrasound irradiation, resulting in outstanding product yields ranging from 90 % to 99 %, as detailed in Scheme 32. The methodology proposed by the group is distinguished by its operational simplicity, producing reactions that are not only streamlined and clean but also characterized by short reaction durations and excellent product yields, thereby advancing atom economy. Furthermore, the avoidance of chromatographic separation techniques underlines the protocol's alignment with green chemistry principles [93].

**Scheme 33:**
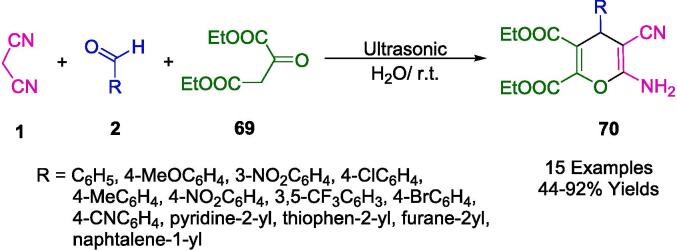
Synthesize 4*H*-pyrans.

By condensation of aromatic aldehydes (**2**), malononitrile (**1**), and enol derivative (**69**) in the presence of triethylamine (Scheme 33), Herrera *et al.* developed a simple and sustainable one-pot protocol using ultrasound to obtain highly substituted 4*H*-pyran structures (**70**) for biological scaffolds. Short reaction times durations, clean procedures, and high product yields make this operating technique very simple straightforward [94].

The investigation into the DNA binding affinity of the synthesized 4*H*-pyran compounds was meticulously conducted using a suite of analytical techniques including viscosity measurements, circular dichroism, UV–visible absorption, and fluorescence spectroscopy. The collected data reveal that these 4*H*-pyran molecules associate with DNA predominantly through interactions with the minor groove, as opposed to intercalative binding or engagement with the major groove. Notably, the binding constant (Kb) for these interactions was found to exceed those documented in prior studies, indicating a strong and specific binding affinity. This comprehensive analysis marks the original report on the interactions between pyrans and their DNA receptors, unveiling a novel area of study. The implications of these findings suggest that such 4*H*-pyran structures could be further refined and potentially harnessed as promising candidates in the development of new therapeutic drugs, given their distinctive DNA binding properties.

**Scheme 34:**
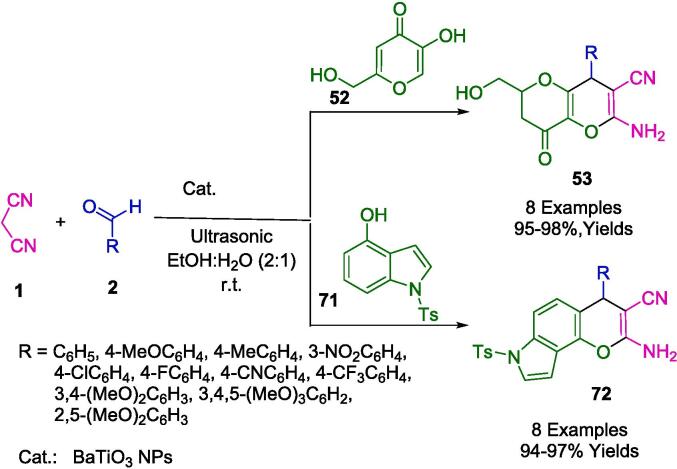
Synthesis of pyrano[3,2-*b*]pyran and 7-tosyl-4,7-dihydropyrano[2,3-*e*]indole derivatives.

Ramana and colleagues advanced the field of nanocatalysis by synthesizing Barium titanate nanoparticles (BaTiO NPs), showcasing their application as an efficient and recyclable heterogeneous catalyst. This innovative catalyst was utilized in the synthesis of pyrano[3,2-*b*]pyran (**53**) and the novel compound 7-tosyl-4,7-dihydropyrano[2,3-*e*]indole (**72**). The synthetic process involved a reaction mixture of an aromatic aldehyde (**2**), malononitrile (**1**), and either kojic acid (**52**) or 1-tosyl-1*H*-indol-4-ol (**71**) in ethanol:water solution (2:1) contained within a round-bottom flask. An ultrasonic horn was employed as the energy source for the reaction, and ethanol:water (2:1) served as the green solvent in accordance with Scheme 34. When subjected to room-temperature ultrasonication, the process yielded the desired products with impressive efficiency, achieving yields between 94 and 98 %. Furthermore, the BaTiO NPs catalyst displayed remarkable durability, remaining active over the course of five consecutive reaction cycles without loss of efficacy. The synthesis, thus, stands out for its operational ease, high product yields, expeditious reaction times, and simple purification process [95].

**Scheme 35:**
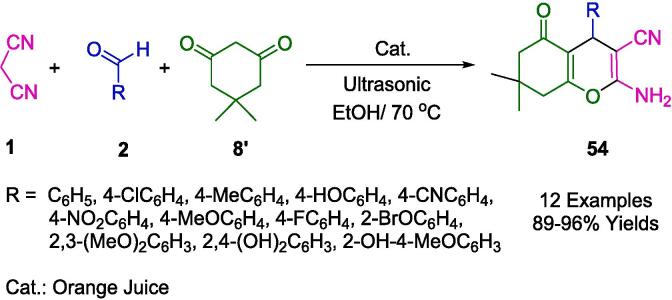
Synthesis of 4*H*-chromene-3-carbonitrile derivatives.

Pansare and colleagues adeptly utilized ultrasonic energy to facilitate the synthesis of 4*H*-benzopyren derivatives (**54**), employing a green synthesis approach by using orange extract in ethanol as an innovative and cost-effective catalytic system (as delineated in Scheme 35). The synthesis was carried out at room temperature, mixing aldehydes (**2**), malononitrile (**1**), and dimedone (**8′**) in ethanol, with the orange extract acting as a natural catalyst under the influence of ultrasound irradiation. The process not only afforded the corresponding 4*H*-chromene-3-carbonitrile compounds in excellent yields but also impressively reduced the reaction times. Furthermore, these synthesized compounds were rigorously tested for biological activity using the 'HRBC membrane stabilization method', and they demonstrated promising anti-inflammatory properties in vitro, indicating their potential for further development as therapeutic agents [96].

**Scheme 36:**
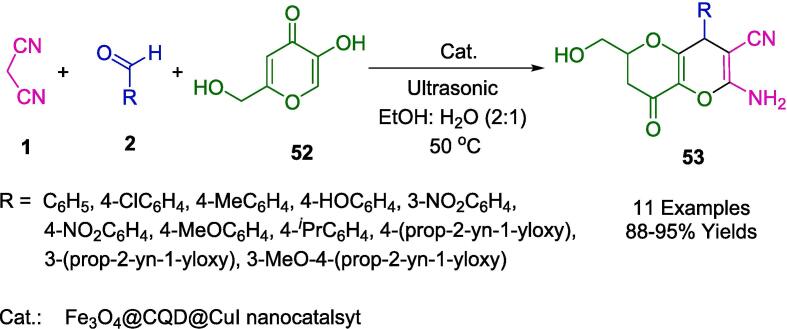
Synthesis of dihydropyrano[3,2-*b*]pyrans.

The role of magnetic nanoparticles in heterogeneous catalysis has been reported in multiple studies cited throughout this review. A series of dihydropyrano[3,2-*b*]pyran derivatives (**53**) were synthesized by Najafi *et al.* using a green ultrasound-assisted method by combining kojic acid (**52**), malononitrile (**1**), and aromatic aldehydes (**2**) in EtOH with water in a mixture of 2:1. Using a magnetic nanocatalyst, carbon quantum dots and copper (I) iodide (Fe_3_O_4_@CQD@CuI) were used as eco-friendly heterogeneous Lewis/Bronsted acid sites and Cu (I) nanocatalysts (Scheme 36). The reactions were sonicated at 50 °C. No noticeable degradation in the catalytic efficiency was observed in a cycle of six reactions for this nanocomposite [97].

**Scheme 37:**
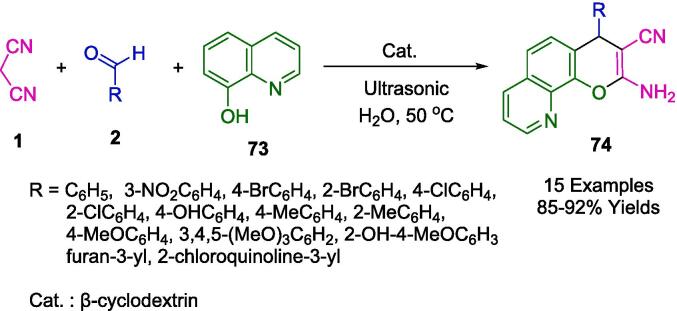
Synthesis of 2-amino-4*H*-pyranoquinoline frameworks.

Through three-component MCR mediated by condensation of aromatic aldehydes (**2**) with malononitrile (**1**), the Gill group synthesized a series of substituted 2-amino-4*H*-pyranoquinoline heterocycles (**74**) using 8-hydroxyquinoline (**73**) catalyzed by *β*-cyclodextrin as a reusable supramolecular catalyst in an aqueous medium under ultrasound irradiation (Scheme 37). The reaction was performed in an ultrasonic bath at 50 °C. Pharmaceutical chemistry requirements were met by synthesizing the desired products in tandem [98].

**Scheme 38:**
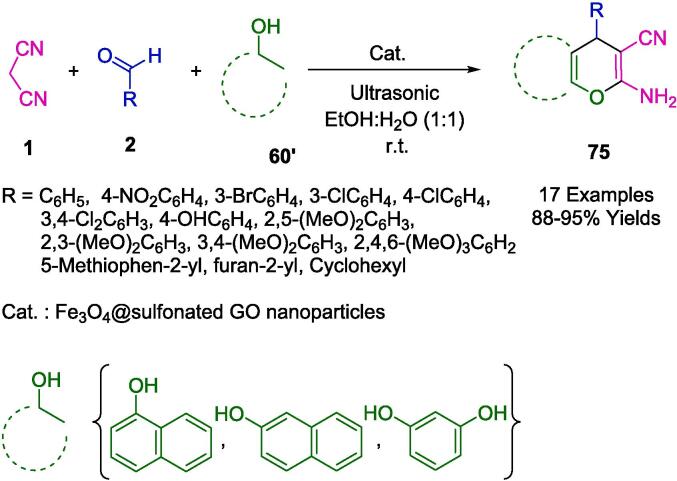
Synthesis of 2-Amino-3-cyano-4*H*-chromene frameworks.

Using Fe_3_O_4_-supported sulfonated graphene oxide as a green and magnetically separable nanocatalyst (Scheme 38), Sharma's group developed a highly convenient and sustainable ultrasonic irradiation domino Knoevenagel–Michael condensation protocol for the synthesis of medicinally advantageous 2-Amino-3-cyano-4*H*-chromene derivatives (**75**). The reaction involves readily available carbonyl compounds, such as aldehydes, ketone esters, and a-naphthol/b-naphthol/resorcinol in 1:1 solutions of H_2_O and EtOH. There was little loss in catalytic activity when the organocatalyst was reused for up to five runs. There are many reasons to prefer the current protocol, including the high atom economy (95 %), excellent yields (88–95 %), short reaction times, waste-free conditions, cost-effectiveness, the use of a non-toxic solvent that requires no reflux temperature, non-chromatographic product purification, and the possibility of recyclability of catalysts [99].

To study docking interactions with the selected proteins DNA gyrase (1KZN) and CYP51 (4WMZ), the highest docking scores were 4 h (-8.8 kcal/mol) and 4e (-10.1 kcal/mol). Toxicity and ADME analyses were also performed on docked compounds and reference drugs.

**Scheme 39:**
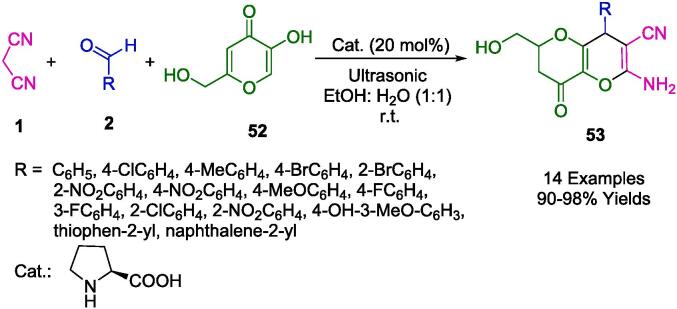
Synthesis of amino substituted-2,4-dihydro-pyrano[3,2-*b*]pyran-3-carbonitrile derivatives.

Through MCR, the Chowhan group synthesized amino-substituted 4,8-dihydropyrano[3,2-*b*]pyran-3-carbonitrile derivatives (**53**) that are medicinally privileged (Scheme 39). Under ultrasound irradiation in an aqueous ethanolic solution at ambient temperature, aryl aldehydes (**2**), malononitrile (**1**), and kojic acid (**52**) are used as starting materials for a domino three-component reaction, and *L*-Proline as secondary amine catalyst (Scheme 21). The reaction was performed in an ultrasonic bath at room temperature. As a scale-up technique and for other valuable transformations, this method was highly efficient. In addition to mild reaction conditions, energy efficiency, short reaction times, fast reactions, simple work-up procedures, broad tolerances for functional groups, a reusable catalyst, a green solvent system, being metal-free, ligand-free, and inexpensive, the methodology provides a number of significant advantages. Despite the absence of chromatography, excellent chemical yields were achieved [100].

**Scheme 40:**
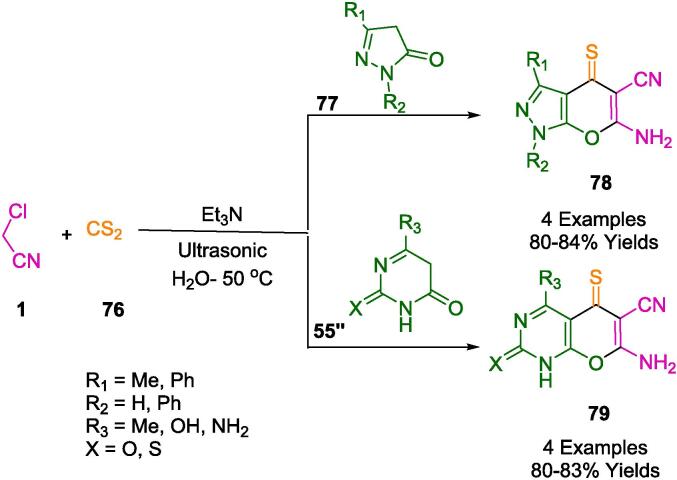
Synthesis of 1,4-dihydropyrano[2,3-*c*]pyrazole-5-carbonitrile derivatives, 5-thioxo-pyranopyrimidine, and 5-thioxopyridopyrimidine systems.

Ali *et al.* have formulated a straightforward and eco-friendly synthetic route to create a new class of compounds: 6-amino-4-thioxo-pyrano[2,3-*c*]pyrazole-5-carbonitriles (**78**), 7-amino-5-thioxo-pyrano[2,3-*d*]pyrimidine-6-carbonitriles, and 4,7-diamino-5-thioxo-pyrido[2,3-*d*]pyrimidine-6-carbonitriles (**79**). This synthetic approach capitalizes on readily accessible reactants, including carbon disulfide (**76**), malononitrile (**1**), and heterocyclic compounds with active methylene groups (**77**, **55′'**), utilizing triethylamine as a catalyst within an aqueous medium enhanced by ultrasonic energy. The methodology is distinguished by its expedited reaction times and the generation of the target molecules in good to excellent yields as outlined in Scheme 40. The process eschews the need for post-reaction chromatographic purification, bolstering its environmental credentials. Comprehensive characterization of the novel compounds was achieved through several analytical and spectroscopic techniques, confirming their structures and purities [76].

**Scheme 41:**
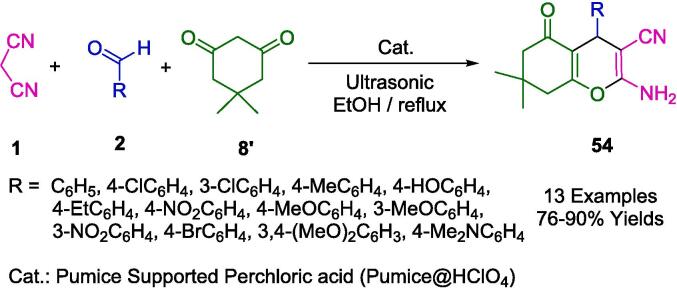
Synthesis of tetrahydrobenzo[*b*]pyran derivatives.

A novel heterogeneous pumice-supported perchloric acid was synthesized by Shirole's group in connection with the development of efficient, modern, and ecological catalytic systems. This was used for the sonochemically synthesizing tetrahydrobenzo[*b*]pyran derivatives (**54**) by combining aldehyde (**1**), malononitrile (**1**), and dimedone (**8′**) in ethanol medium (Scheme 41). A reflux ultrasound cleaning bath was used to investigate the reaction's recyclability and reusability with pumice-supported perchloric acid catalysts. Despite reusing the catalyst at least three times with no evidence of loss of catalytic efficiency, the catalyst was recovered quantitatively by simple magnetic separation. There are numerous advantages to the present protocol, including the one-pot reaction, good yield, short reaction time, inexpensive catalyst, recyclability and reusability of the catalyst, simplicity in experimental and work-up procedures, and the possibility of purifying targeted molecules without column chromatography [101].

**Scheme 42:**
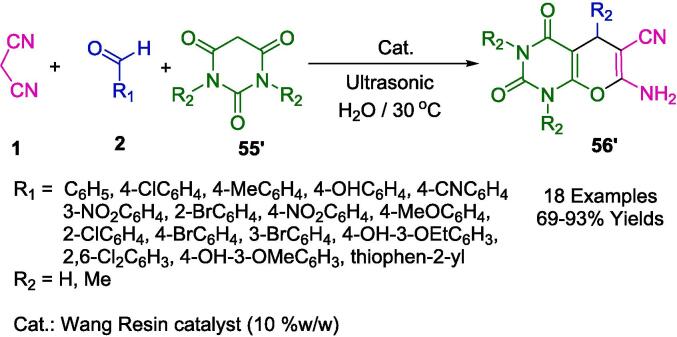
Synthesis of pyrano[2,3-*d*]pyrimidine-2,4(3*H*)-dione derivatives.

Srimannarayana and co-workers developed a greener MCR for the ultrasound-mediated (US) preparation of a series of synthesized pyrano[2,3-d]pyrimidine-2,4(3*H*)-dione derivatives (**56′**) for their potential inhibitory properties against SIRT1 (Sirtuin 1) (Scheme 42). Rapid access to this class of compounds was achieved via a sonochemical approach involving the Wang-OSO_3_H catalyzed three-component reaction condensation of barbituric acid (**55**), aromatic aldehyde (**2**), and malononitrile (**1**) in water at 30 °C temperature in good to acceptable yields.

In silico, several compounds were initially evaluated as potential hits against SIRT1 (Sirtuin 1). Additionally, docking some of them into hSIRT2 demonstrated that they have weaker interactions with this protein, indicating that they prefer SIRT1 to SIRT2. When tested in vitro against SIRT1, most synthesized compounds showed a greater than 50 % inhibition. The most potent compound among all those tested, 7-amino-5-(3-bromophenyl)-2,4-dioxo-1,3,4,5-tetrahydro-2*H*-pyrano[2,3-*d*]pyrimidine-6-carbonitrile, was several times more potent than nicotinamide based on SIRT1 IC_50_ values. The in-silico assessment also predicted that it would have favorable pharmacokinetic properties in MCF7 and HEK 293 T cells [102].

### 6.2 Pyrano-pyrazolone derivatives

**Scheme 43:**
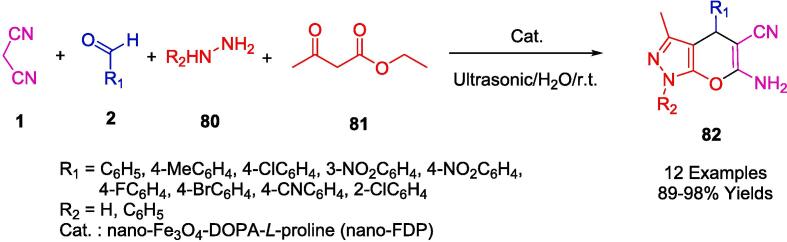
Synthesis of pyrano-pyrazolone derivatives.

In a related investigation into pyranopyrazolone structures, the research conducted by Pal and colleagues culminated in the development of notably robust, superparamagnetic nanoparticles that supported *L*-Proline catalysis (referred to herein as nano-FDP). This study harnessed the virtues of cost-effective and readily procurable reactants. A novel synthesis approach was employed wherein ethyl acetoacetate (**81**), hydrazine hydrate (**80**), diverse aldehydes (**2**), and malononitrile (**1**) were reacted in an aqueous medium at ambient temperature, as delineated in Scheme 43. Noteworthy is the implementation of ultrasonication at room temperature—a technique that significantly expedited the synthesis of the pyran derivatives. The outcomes of this method were remarkable, achieving not only a high yield of the targeted compound but also presenting an eco-friendly aspect by utilizing water as the solvent. This greener approach further allowed for the facile magnetic retrieval of the catalyst, presenting an amalgam of efficiency and sustainability in the synthetic procedure [103].

**Scheme 44:**
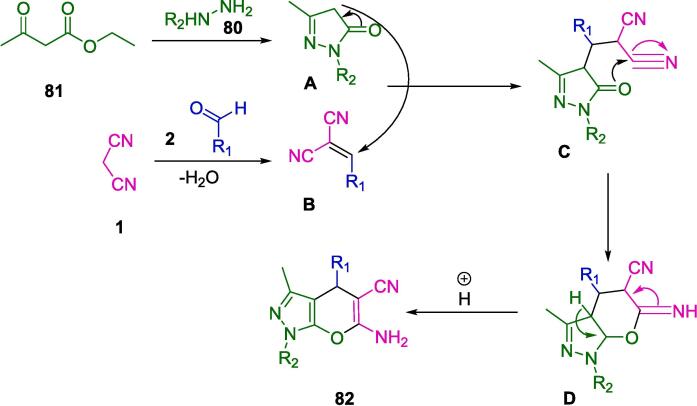
Plausible strategy for synthesizing pyranopyrazolones.

Scheme 44 illustrates a plausible strategy for synthesizing pyranopyrazolones (**74**) with nano-FDP. The reaction between ethyl acetoacetate (**81**) and either hydrazine hydrate (**80**) or phenyl hydrazine (**80′**) produces pyrazolone derivatives (**A**). As a second step, nano-FDP binds to the aldehyde (**2**) to form a cyano-olefin compound (**B**) by combining it with an iminium intermediate (**C**). An intermediate (**C**) is then formed by nano-FDP, which then undergoes intermolecular cyclization to yield the desired product (**74**).

**Scheme 45:**
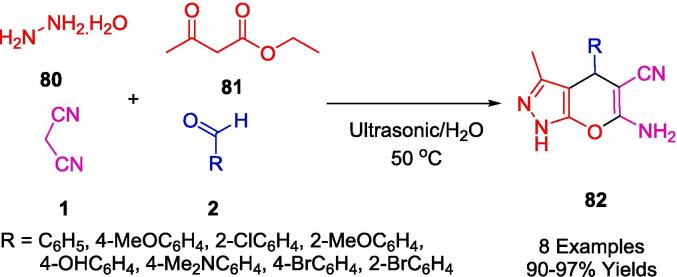
Synthesis of pyranopyrazoles.

Jonnalagadda's group reported the development of a straightforward, catalyst-free, and environmentally friendly multicomponent synthetic approach for the preparation of pyranopyrazoles (**82**) in one pot involving aromatic aldehydes (**2**), hydrazine monohydrate (**80**), ethylacetoacetate (**81**) and malononitrile (**1**) in water, irradiated by ultrasound (Scheme 45). The purpose of this protocol is to avoid conventional chromatography and purification steps and to achieve high levels of selective conversion with no by-products being produced [104].

**Scheme 46:**
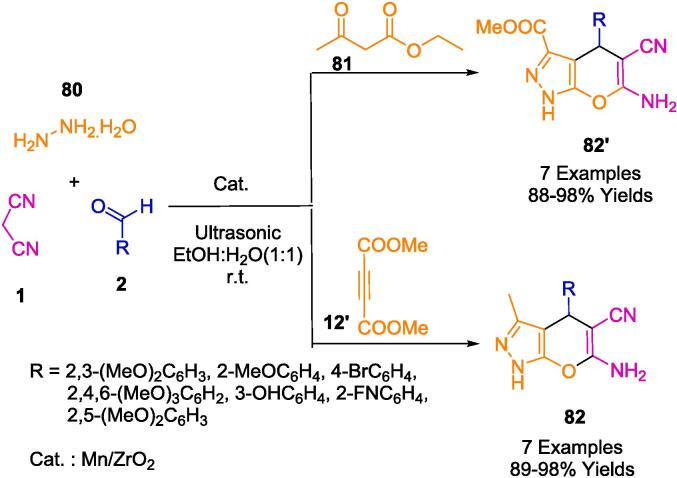
Synthesis of pyrano[2,3-*c*]pyrazoles-3-carboxylate derivatives.

Building upon their assessments in the field of nanocatalysts, Jonnalagadda and colleagues have innovated a recyclable heterogeneous solid catalyst, specifically manganese-supported zirconium (Mn/ZrO_2_). This catalyst was engineered for the eco-conscious fabrication of pyrano[2,3-*c*]pyrazole-3-carboxylate/pyrano[2,3-*c*]pyrazole-5-carbonitrile derivatives (**82**, **82′**), achieving outstanding yields ranging from 88 % to 98 % within abbreviated reaction durations, as described in Scheme 46. Within the ambit of green chemistry, Mn-doped zirconia has been identified as a potent and sustainable catalyst. It facilitates a four-component reaction orchestrated through ultrasonic activation, incorporating reactants such as dimethylacetylenedicarboxylate (**12′**)/ethyl acetoacetate (**81**), hydrazine hydrate (**80**), malononitrile (**1**), and aromatic aldehydes (**2**). The resilience of this nanocomposite is particularly noteworthy; it maintains its activity over six consecutive cycles with a simple recovery protocol that entails ethyl alcohol rinsing and subsequent desiccation. Moreover, the catalyst's regenerative property gives emphasis to its environmental and practical merits, circumventing the necessity for chromatographic purification processes [105].

**Scheme 47:**
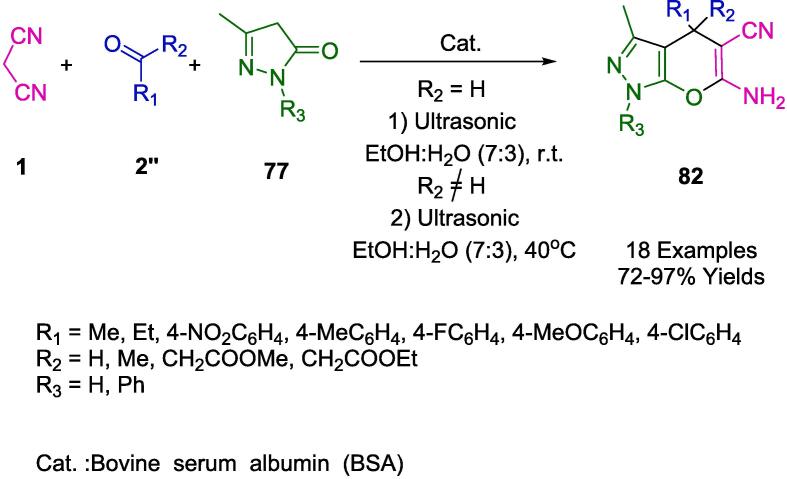
Synthesis of dihydropyrano[2,3-*c*]pyrazole derivatives.

The strategic deployment of biocatalysts for conducting chemical transformations has been affirmed as an approach aligned with the principles of green chemistry. The application of such biocatalysts is laudable for generating minimal by-products, thanks to the high specificity of the reactions, benign reaction conditions, and reduced energy consumption. Chaudhari and colleagues have elucidated the concept of biocatalytic promiscuity, presenting it as an innovative avenue for broadening the utility of enzymes in the realm of organic synthesis. This was exemplified in their work by employing bovine serum albumin (BSA) as a biocatalyst to craft dihydropyrano[2,3-*c*]pyrazole derivatives (**82**) through a one-pot synthesis that is both environmentally congenial and efficient. The methodology, detailed in Scheme 47, involves an ultrasonic-assisted condensation of a variety of aldehydes (**2**) with aromatic/aliphatic ketones (**2′'**), malononitrile (**1**), and 3-methyl-1*H*-pyrazol-5(4*H*) (**77**) in a mixed aqueous-ethanolic medium (H_2_O-EtOH, 7:3) at room temperature. A notable aspect of this biocatalyst is its sustainability, evidenced by its capability to be recycled and retain significant catalytic performance after three consecutive uses. The protocol championed by BSA is not only eco-friendly and excludes toxic solvents but also delivers impressive yields and is straightforward to administer. Consequently, these attributes render BSA an enticing entity for further exploration and potential expansion of its biocatalytic repertoire [106].

**Scheme 48:**
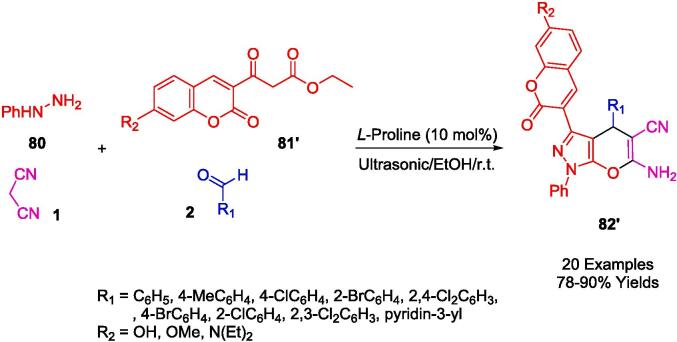
Synthesis of substituted pyrano[2,3-*c*]pyrazole-5-carbonitrile scaffolds.

Employing *L*-Proline as an organocatalyst, the research spearheaded by Ablajan *et al.* has resulted in the formulation of a streamlined and effective protocol for the assembly of dihydropyrano[2,3-*c*]pyrazoles integrated with coumarin motifs (**82′**) as depicted in Scheme 48. This synthesis process, a four-component reaction, is facilitated by *L*-Proline in an ethanol solvent system. The reaction constituents comprising phenylhydrazine (**80**), a diketone (**81′**), a suite of aromatic aldehydes (**2**), and malononitrile (**1**) are subjected to ultrasonic irradiation at ambient temperatures within the ethanol medium. The ultrasonic approach notably yields a spectrum ranging from good to excellent yields of the targeted dihydropyrano[2,3-*c*]pyrazole compounds replete with coumarin structures. The advantages of this technique are multifold: abbreviated reaction times, heightened eco-compatibility, diminished environmental footprint, operational simplicity, and enhanced yield efficiency. This method represents a substantial advancement in the field of green chemistry, integrating organocatalysis with ultrasonication to achieve notable synthetic efficiency [107].

**Scheme 49:**
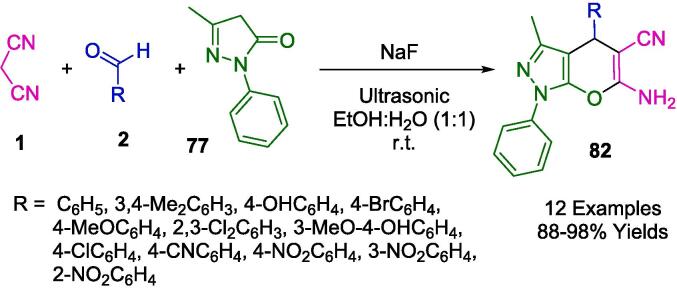
Synthesis of series of dihydropyrano [2,3-*c*]pyrazoles.

In their pursuit to synthesize dihydropyrano[2,3-*c*]pyrazoles (**82**), a compound class noted for their biological efficacy, Kotha and associates unveiled methodologies that leverage ultrasonic irradiation, delineated in Scheme 49. Utilizing ambient temperature conditions coupled with ultrasonication, they facilitated a reaction between 3-Methyl-1-phenyl-2-pyrazoline-5-one (**77**), aromatic aldehydes (**2**), and malononitrile (**1**) within an aqueous methanol solution. Notably, the employment of a fluoride salt catalyst within a solvent system comprising ethanol and water in equal parts was pivotal in the successful synthesis of a diverse series of these compounds. This newly developed protocol holds an edge over its predecessors, primarily due to the cost-effectiveness and efficiency of the catalyst, the mildness of the reaction conditions, the streamlined workup procedure, the reduced reaction durations, and the augmented yield potentials [108].

**Scheme 50:**
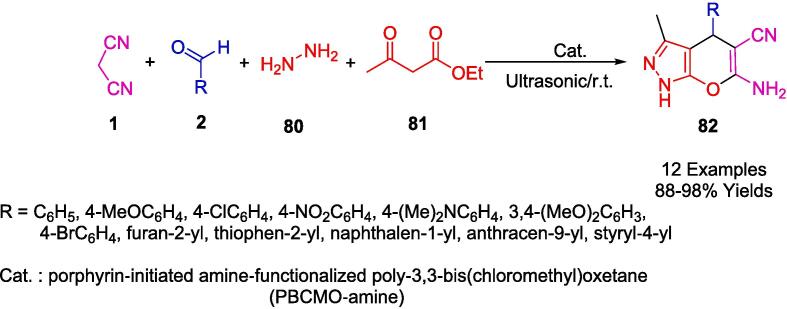
Synthesized pyranopyrazole derivatives.

Krishnapillai *et al.* have innovatively synthesized a new porphyrin-initiated, amine-functionalized poly-3,3-bis(chloromethyl)oxetane (PBCMO-amine) dendritic polymer, which they subsequently employed as a heterogeneous nanocatalyst in the synthesis of pyranopyrazole derivatives (**82**), as detailed in Scheme 50. The synthesis involved a multicomponent reaction (MCR) that took advantage of a solvent-free environment at ambient temperatures, using substituted benzaldehyde (**2**), malononitrile (**1**), ethyl acetoacetate (**81**), and hydrazine hydrate (**80**) as reactants, with ultrasonication facilitating the process. The ultrasonic bath was a critical component in achieving the high purity and yield of the resulting compounds, simplifying the purification process to mere re-crystallization, thus obviating the need for chromatographic separation techniques. The nanocatalyst's water-solubility conferred an additional benefit, allowing for its straightforward separation and consequent reuse. Remarkably, this catalyst retained its catalytic prowess for up to five consecutive cycles without any significant diminution in activity [109].

### 6.3 *S*-Heterocyclic compounds

**Scheme 51:**
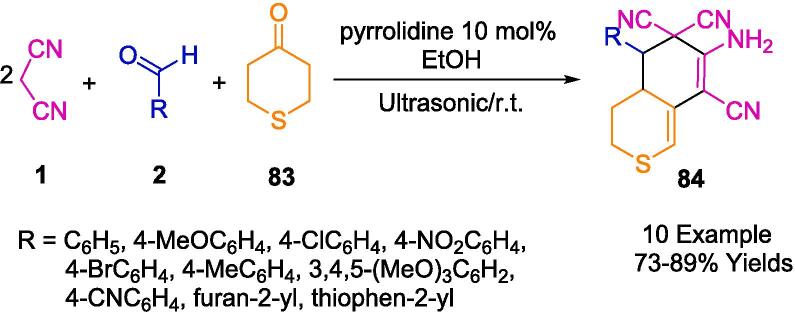
Synthesized tetrahydro-6*H*-isothiochromene-6,6,8-tricarbonitrile derivatives.

Mojtahedi *et al.* reported a cost-effective, highly useful, and eco-friendly aqueous MCR procedure for the one-pot synthesis of highly substituted tetrahydro-6*H*-isothiochromene-6,6,8-tricarbonitrile derivatives (**84**) from various aldehydes (**2**), two equivalent malononitrile (**1**) and thiopyran-one (**83**) (Scheme 51). The reactions were performed in an ultrasound bath in 10 mol% pyrrolidine in ethanol at room temperature. Green solvents make this methodology very interesting from an economical and environmental perspective. This method provides several advantages such as operational simplicity, the use of accessible and economical starting materials, and reduced environmental consequences. Through simple filtration and recrystallization of precipitated solids, we were able to avoid cumbersome and expensive chromatographic separations [110].

**Scheme 52:**
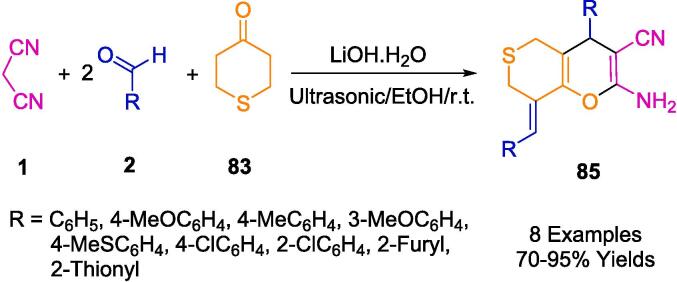
Synthesis of derivatives of thiopyrano[4,3-*b*]pyran structures.

In another study, Mojtahedi et al. developed an efficient one-pot procedure for the synthesis of a series of derivatives of the thiopyrano[4,3-*b*]pyran structure (**85**) that is sonocatalyzed by quantities of LiOH·H_2_O (Scheme 52). It was found that thiopyrano[4,3-*b*]pyran compounds (**85**) could be synthesized at room temperature in ethanol under mild conditions using tetrahydro-4*H*-thiopyran-4-one (**83**), aromatic aldehydes (**2**), and malononitrile (**1**) as starting materials. Separating products from reaction mixtures requires no cumbersome and expensive chromatographic separation because spontaneous precipitation occurs naturally in reaction mixtures [111].

**Scheme 53:**
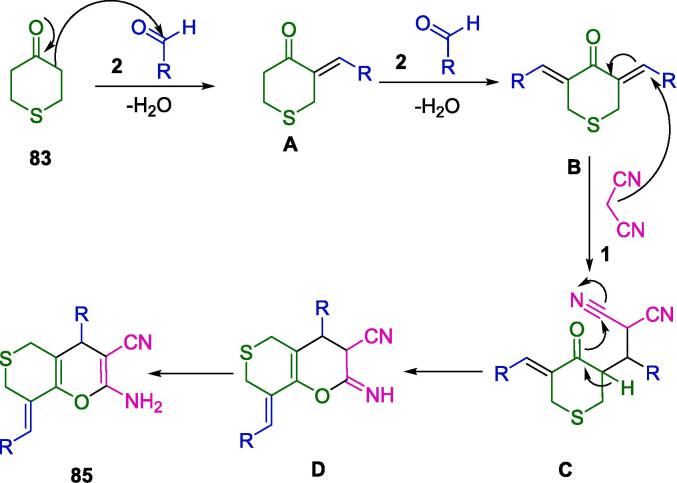
Plausible mechanism for synthesis of thiopyrano[4,3-*b*]pyran structures.

Bisarylmethylidene intermediate **B** is formed by double-aldol condensation during the initial reaction (Scheme 53). A Michael addition with malononitrile (**1**) is carried out in situ, followed by a nucleophilic cyclization. After sonication, the expected product derivative (**46**) is formed.

### 6.4 Spiro heterocycles

**Scheme 54:**
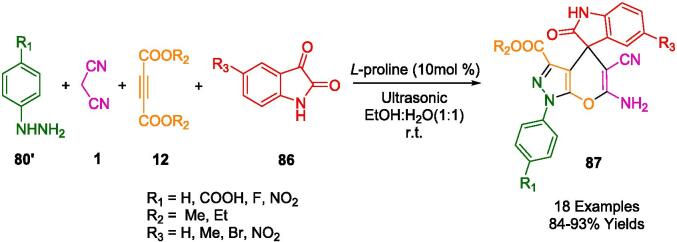
Synthesis of spiro[indoline-3,4′-pyrano[2,3-*c*]pyrazole] derivatives.

The unique architecture of spiro-oxindoles has established them as focal structures within natural products and pharmaceutical compounds, as referenced in literature [112], [113], [114], [115]. The quest for environmentally benign synthetic strategies to craft these compelling molecules is a topic of considerable interest. In a notable advancement, spiro-oxindoles have been proficiently synthesized via a one-pot methodology that integrates ultrasound-assisted synthesis with organocatalysis. In this context, Ablajan and his team have harnessed the catalytic prowess of *L*-Proline, an amino acid derivative, to facilitate the formation of Spiro[indoline-3,4′-pyrano[2,3-*c*]pyrazole] derivatives (**87**). The reaction milieu was an equimolar water:ethanol solvent system. This synthesis was achieved by orchestrating the condensation of substituted phenylhydrazine (**80′**) with dialkylacetylenedicarboxylate (**12**), in concert with substituted isatin (**86**) and malononitrile (**1**), all at ambient temperatures as delineated in Scheme 54 [116].

**Scheme 55:**
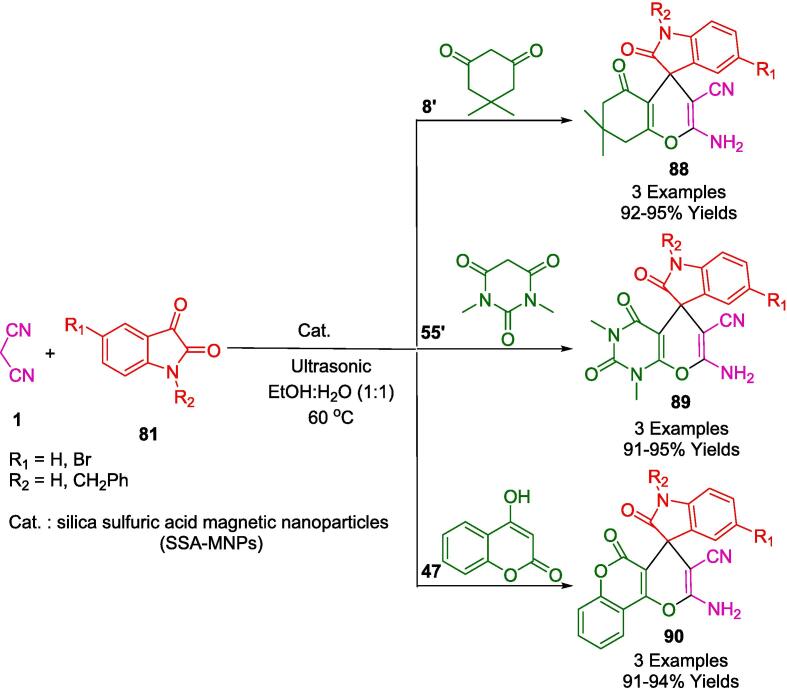
Synthesis of Spiro[2-amino-4*H*-pyran-oxindole]s.

In their quest to develop sustainable chemical synthesis, Karimi *et al.* discovered an effective heterogeneous nano-catalyst made up of silica sulphuric acid (SSA) magnetic nanoparticles. This catalyst was found to be instrumental in the ultrasound-assisted synthesis of Spiro[2-amino-4*H*-pyran-oxindole] derivatives (**88**, **89**, **90**), as detailed in Scheme 55. The catalytic activity of SSA-MNPs was harnessed for a three-component reaction involving isatins (**86**), malononitrile (**1**), and an additional component chosen from dimedone (**8′**), 1,3-dimethylbarbituric acid (**55′**), or 4-hydroxycoumarin (**47**). The reactions were conducted at a controlled temperature of 60 °C, culminating in high to excellent yields and endorsing a clean, eco-friendly synthetic pathway. A prominent feature of the SSA-MNPs catalyst is its ease of recovery from the reaction milieu, achieved through the simple application of an external magnetic field. However, it is noteworthy that the catalyst's performance diminishes after four cycles, indicating a limitation in its reusability. Despite this, the protocol described offers numerous advantages: it operates under comparatively mild conditions, achieves rapid reaction completion, ensures high yields of the desired products, and the catalyst can be reused multiple times, which is a greener alternative compared to conventional homogeneous acid catalysts. This innovative approach thus represents a valuable contribution to green chemistry, facilitating more sustainable and environmentally considerate chemical processes [117].

**Scheme 56:**
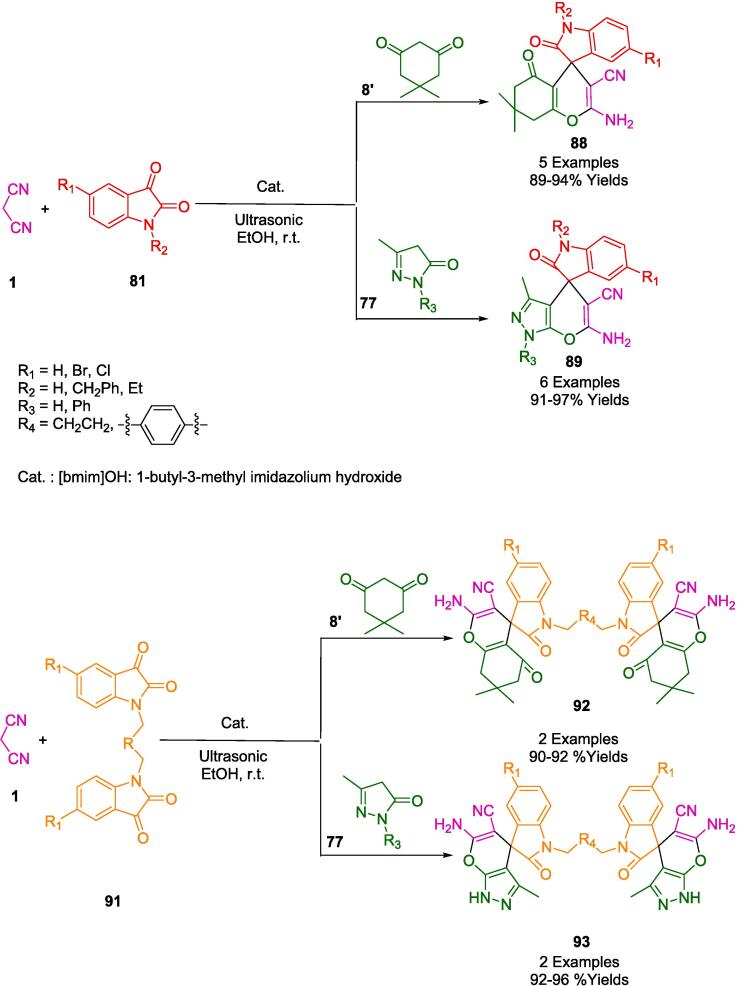
Synthesis of spirooxindole derivatives.

Padvi *et al.* utilized a rapid and efficient one-pot method to synthesize a series of spiro oxindole derivatives (**88**, **89**) through a three-component reaction involving isatin (**86**), malononitrile (**1**), and a carbonyl compound with a reactive α-methylene group (**8′**), (**77**). The reaction was catalyzed by the task-specific ionic liquid, 1-butyl-3-methyl imidazolium hydroxide [bmim]OH, as shown in Scheme 56. This protocol proved to be more efficient than some of the previous methods for the synthesis of benzimidazoloquinazolines reported in the literature, revealing advantages regarding catalyst loading, solvent, short reaction times at room temperature, and the absence of hazardous organic solvents, toxic catalysts, and tedious purification processes. In this protocol, new bis-Spiro oxindole systems (**85**, **86**) are synthesized as well as mono-Spiro oxindoles. There is a straightforward way to separate the reusability of the product from the catalyst with excellent yields. Up to five times of reuse without loss of activity was possible with the [bmim]OH catalyst system [118].

**Scheme 57:**
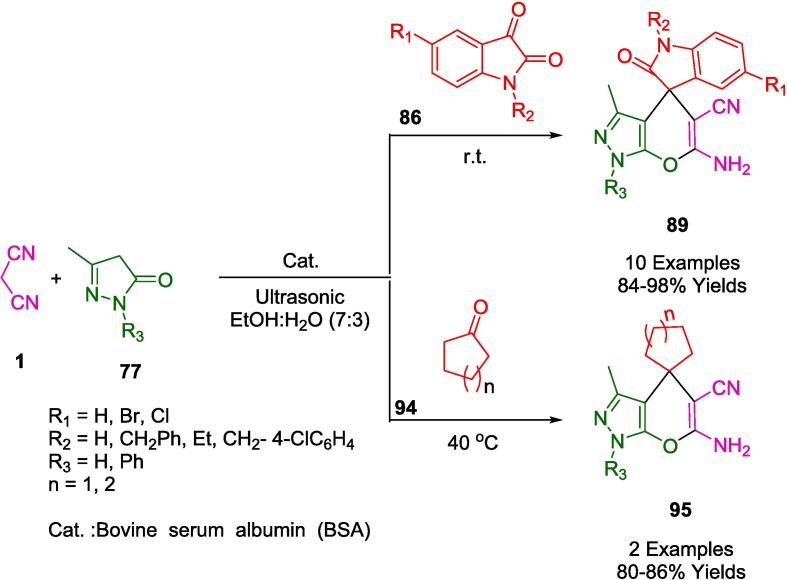
Synthesis of spiro[substituted-1,4′-pyrano[2,3-*c*]pyrazoles.

Chaudhari *et al.* have developed a green one-pot method for synthesizing Spiro[indoline-3,4′-pyrano[2,3-*c*]pyrazole] derivatives (**89**, **95**) using bovine serum albumin (BSA) (Scheme 57). The protocol involves a three-component condensation in EtOH:H_2_O (7:3) utilizing isatin (**86**), cyclopentanone, or cyclohexanone (**94**), malononitrile (**1**), and 3-methyl-1*H*-pyrazol-5(4*H*)one (**77**), with ultrasound assistance. Biocatalysts can be reused three times without significant catalytic activity loss. The applications of biocatalysts for BSA can be further expanded due to eco-friendliness, reusability, low solvent usage, excellent yields, ease of workflow, and lack of byproducts [106].

**Scheme 58:**
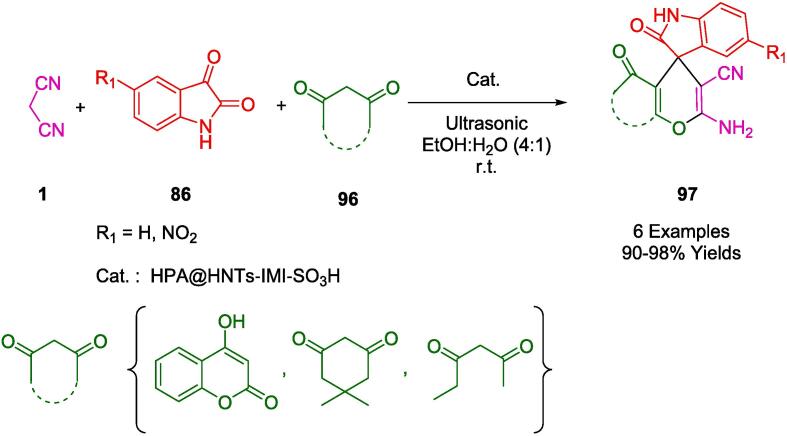
Synthesis of spirooxindole derivatives.

Heravi and colleagues have shown a remarkable dedication to environmental sustainability in synthetic chemistry through the use of a heterogeneous nano catalyst, HPA(HNTs)-IMI(SO_3_H), for the high-performance synthesis of the spirooxindole derivative (**97**). The state-of-the-art catalyst in question was ingeniously crafted by modifying halloysite nanotubes with an integrated ionic liquid framework and amalgamating a heteropolyacid. This catalytic system is meticulously designed to leverage the benefits of ultrasonic energy to expedite reaction kinetics, thus facilitating the condensation of isatins (**86**) with malononitrile (**1**) and 1,3-dicarbonyl compounds (**96**) to yield the targeted Spiro oxindole derivatives, as explicated in Scheme 58. Notably, the nanocomposite catalyst showcases an impressive reusability profile, retaining its catalytic efficiency across three successive reactions without any detectable decline in performance. This characteristic is particularly advantageous, aligning with the principles of green chemistry by reducing waste and avoiding the frequent need for catalyst replacement. The combination of ultrasonic assistance and the robustness of the nanocatalyst provides an exemplary model of innovation in the pursuit of sustainable and environmentally benign chemical processes [119].

**Scheme 59:**
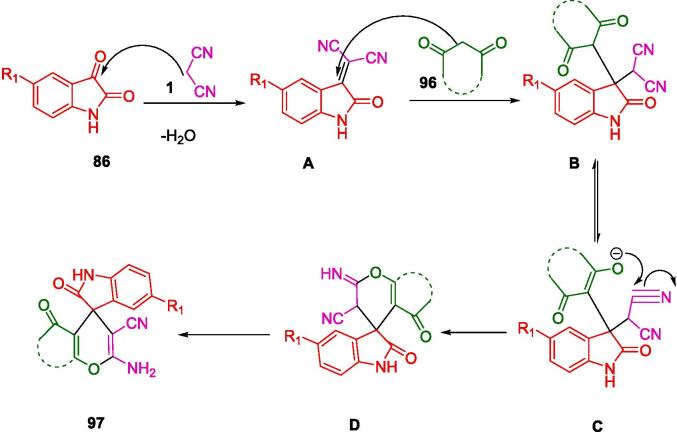
The plausible mechanism of synthesis of Spiro oxindole.

The mechanism presented in Scheme 59 is plausible. Knoevenagel condensation between isatin (**86**) and malononitrile (**1**) is hypothesized to be facilitated by the catalyst. Carbonyl groups in isatin (**86**) are activated by this process. Once the intermediate (**A**) is formed, it reacts with 1,3‐dicarbonyl compounds (**96**) to produce the tolerated intermediate (**C**). The final product (**9**7) is achieved by an intermolecular cyclization event on the intermediate (**C**).

**Scheme 60:**
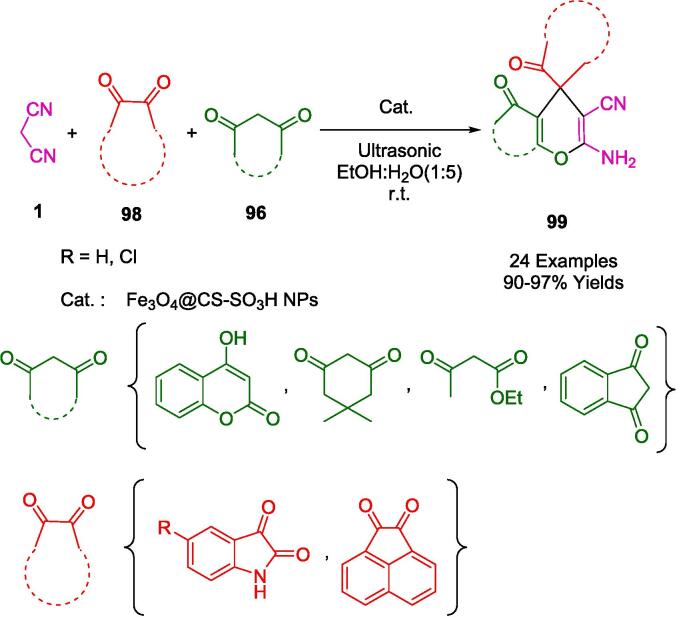
Synthesis of Spiro oxindoles.

Several biological properties are associated with spiro-4*H*-pyrans, which are of particular interest in medicinal chemistry. In green organic synthesis, carbonaceous solids (CSO_3_H) have emerged as heterogeneous catalysts that combine acid-catalytic properties with greater recycling efficiency. From this perspective, Naeimi *et al.* presented a convenient one-pot protocol for the synthesis of spiro-4*H*-pyran frameworks (**99**) using sulfonated chitosan-coated Fe_3_O_4_ nanoparticles (Fe_3_O_4_@CS-SO_3_H NPs) as an efficient nanocatalyst under ultrasonic conditions (Scheme 60). In the presence of Fe_3_O_4_@CS-SO_3_H NPs, isatin (**86**) or acenaphthoquinone (**98**) was condensed in an aqueous ethanol media (5:1) at room temperature with malononitrile (**1**), and different 1,3-dicarbonyl compounds (**96**), in aqueous ethanol media (5:1). Up to five reactions could be carried out with the catalyst after it was filtered and washed. However, the surface of the catalyst, was contaminated with active spits, reducing its catalytic efficiency [120].

**Scheme 61:**
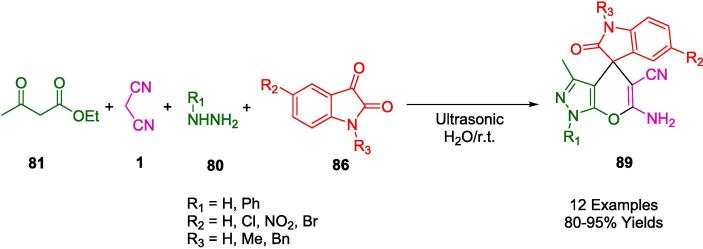
Synthesis of spiro[indoline-3,4′-pyrano[2,3-*c*]pyrazole derivatives.

Hojati *et al.* continued their search for catalyst-free synthetic protocols that were more sustainable. As described, the four-component synthesis of Spiro indoline-3,4′-pyrano[2,3-*c*]pyrazoles (**89**) was accomplished through ultrasonic irradiation in an aqueous medium with condensation of ethyl acetoacetate (**81**) in water with hydrazine hydrate (**80**) or phenylhydrazine (**80′**), isatin (**86**), and malononitrile (**1**) at room temperature at high yields (Scheme 61). In addition to excellent yields, high purity, short reaction times, and the use of water as the solvent, the new method is environmentally friendly [121].

**Scheme 62:**
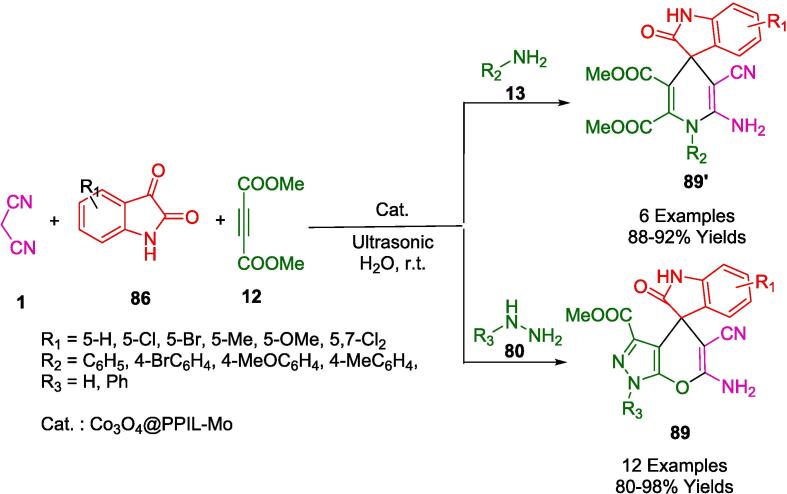
Synthesis of spiropyrano[2,3-*c*]pyrazole carboxylate derivatives and Spiro[indoline-3,4′-pyridine] derivatives.

The research group led by Safaei Ghomi has introduced a green synthetic strategy that involves the encapsulation of cobalt oxide nanoparticles within a polymer matrix of poly (1-aminopropyl-3-vinylimidazolium bromide). Their method facilitates the condensation of isatins (**86**), malononitrile (**1**), and dimethylacetylene dicarboxylate (DMAD) with various amine components, including hydrate hydrazine (**80**), phenylhydrazine (**80**′), and aromatic amines (**13**), under benign conditions. This innovative approach yields spiropyrano[2,3-*c*]pyrazole carboxylate derivatives (**89**) and spiro[indoline-3,4′-pyridine] derivatives (**89′**), as outlined in Scheme 62 [122].

Additionally, a molybdenum Schiff base complex immobilized on a porous polyionic liquid (PPIL) framework was employed as a nanocatalyst, which was activated via sonication in an ultrasonic bath at ambient temperature, with water serving as an eco-friendly solvent medium. The synthesis, conducted under such conditions, is in harmony with the principles of green chemistry, emphasizing the reduction of harmful substances and energy efficiency. Post-synthesis, the nanocomposite catalyst can be efficiently recovered through filtration and subsequent washing, maintaining its catalytic activity for up to three reaction cycles. This reusability, coupled with the solvent's green credentials, demonstrates a high degree of compatibility with green chemistry's objectives, presenting an advantageous route for the synthesis of complex organic compounds with reduced environmental impact.

**Scheme 63:**
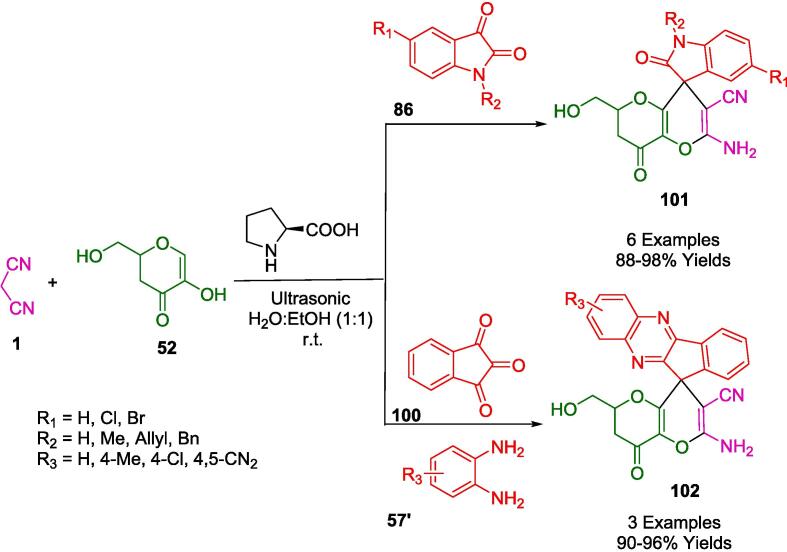
Synthesis of spiro-[indeno[1,2-*b*]quinoxaline-11,4′-pyrano[3,2-*b*]pyran].

The research conducted by Chowhan's team has resulted in the establishment of an eco-friendly and efficient one-pot synthesis for a series of complex spiro compounds, notably spiro-pyrano[3,2-*b*]pyran (**1 0 1**) and spiro-[indeno[1,2-*b*]quinoxaline-11,4′-pyrano[3,2-*b*]pyran] (**1 0 2**). Utilizing *L*-Proline as a secondary amine organocatalyst, the group has successfully orchestrated a domino multicomponent reaction under ultrasonic irradiation within a mixed solvent system of water and ethanol (1:1, v/v). The substrates for these reactions included kojic acid (**52**), malononitrile (**1**), varied substituted isatins (**86**), ninhydrin (**1 0 0**), and *o*-phenylenediamine (**57′**), as presented in Scheme 63. An essential aspect of this methodology is the catalytic longevity of *L*-Proline, which maintained its reactivity over four consecutive cycles with negligible depletion in its efficacy. The developed protocol offers a plethora of advantages, which are pivotal to sustainable chemistry practices. These include the operation under gentle reaction conditions, energy conservation, expeditious reaction kinetics, a simple post-reaction purification process, and compatibility with a wide range of functional groups. The process is distinguished by its metal-free and ligand-free nature, absence of waste by-products, and cost-effectiveness. The avoidance of column chromatography for product purification underscores the method's alignment with the tenets of green chemistry, culminating in high-yielding syntheses that are beneficial from both an environmental and a pharmaceutical perspective [100].

**Scheme 64:**
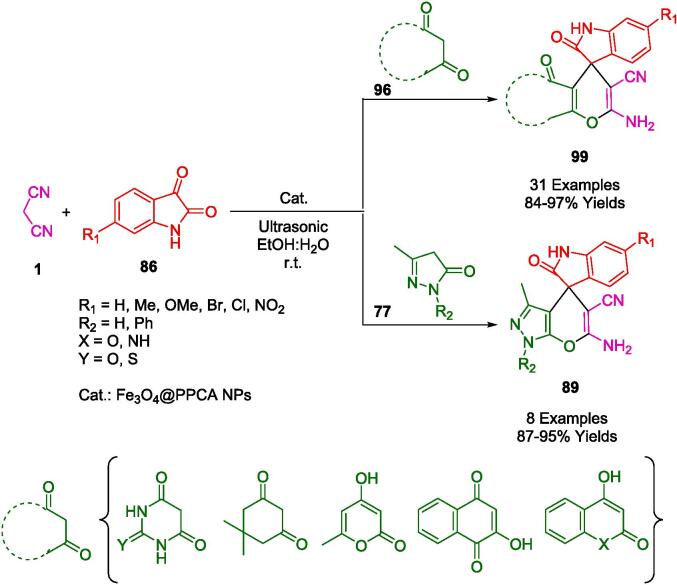
Synthesis of spirooxindole derivatives.

Nongkhlaw *et al.* have contributed to the field of green chemistry by developing a multicomponent reaction (MCR) that effectively synthesizes spirooxindole derivatives (**99**, **89**) via an ultrasound-mediated process (Scheme 64). This approach incorporates a heterogeneous nanocatalyst, Fe_3_O_4_@PPCA, which is magnetically retrievable, underscoring the environmentally benign nature of the catalyst in terms of its recovery and potential for reuse. The reaction uses malononitrile (**1**) and isatin (**86**) as core reactants, along with a variety of 1,3-diketones (**96**) such as dimedone, 2,4-dihydroxyquinoline, 2-hydroxy-1,4-naphthoquinone, 4-hydroxycoumarin, barbituric acid, thiobarbituric acid, 4-hydroxy-6-methyl-2-pyrone, 1*H*-pyrazole, and 1-phenyl-pyrazole. The use of an ethanol:water (1:1) mixture as the solvent system aligns with the principles of green chemistry by minimizing the use of toxic organic solvents and allowing the reaction to proceed at room temperature, which conserves energy. The reuse of the Fe_3_O_4_@PPCA nanocatalyst, however, was observed to be suboptimal, with a notable decline in catalytic activity after five consecutive uses. This indicates that while the catalyst can be recovered magnetically, its longevity is limited, and there may be a need for regeneration or further stabilization to enhance its recyclability. The development of this greener MCR demonstrates the continuous efforts in organic synthesis to balance the need for efficient chemical transformations with the imperative to reduce the environmental footprint of chemical processes [123].

Furthermore, the use of ultrasonication further demonstrated the versatility of this protocol in addition to other features such as simple work-up procedure, mild reaction conditions, simple catalyst recovery, short reaction time, and the use of environmentally friendly solvents. In *vitro* biological evaluation of the synthesized compounds also revealed the antitubercular activity of compound 2-amino-6′-bromo-2′,5,10-trioxo-5,10-dihydrospiro[benzo[*g*]chromene-4,3′-indoline]-3-carbonitrile whereas compounds 7′-amino-2,4′-dioxo-2′-thioxo-1′,2′,3′,4′-tetrahydrospiro[indoline-3,5′-pyrano[2,3-*d*]pyrimidine]-6′-carbonitrile, 7′-amino-6-methoxy-2,4′-dioxo-2′-thioxo-1′,2′,3′,4′-tetrahydrospiro[indoline-3,5′-pyrano[2,3-*d*]pyrimidine]-6′-carbonitrile, and 7′-amino-6-nitro-2,4′-dioxo-2′-thioxo-1′,2′,3′,4′-tetrahydrospiro[indoline-3,5′-pyrano[2,3-*d*]pyrimidine]-6′-carbonitrile showed profound antioxidant activity. A computational study on the mechanistic pathway for organic conversions was also conducted, using density functional theory (DFT), to determine the catalytic role of PPCA.

### 6.5 Miscellaneous

**Scheme 65:**
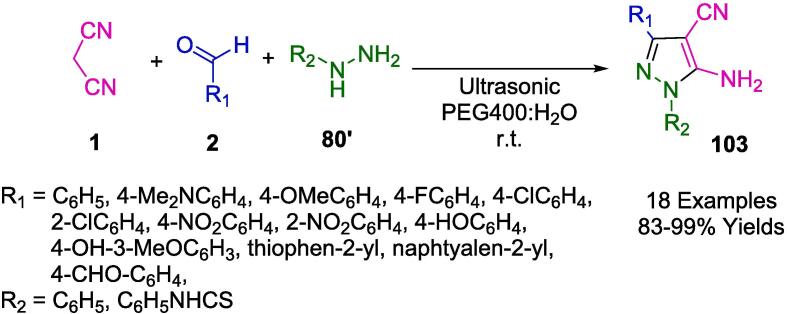
Synthesis of highly substituted pyrazole derivatives.

In their scholarly article, Nemati and colleagues delineated a cost-efficient and environmentally benign multicomponent reaction (MCR) protocol that facilitates the synthesis of densely substituted pyrazole compounds (**1 0 3**). This synthesis is accomplished through a one-pot reaction amalgamating various aldehydes (**2**), malononitrile (**1**), and phenylhydrazine (**80**′), as presented in Scheme 65. Remarkably, the synthesis is conducted in an ultrasonic bath utilizing polyethylene glycol (PEG-400) combined with water at ambient temperature. The absence of a catalyst and the employment of green solvents underpin the economic and environmental allure of this process. The advantages of this synthesis method are manifold, including procedural ease, the utilization of readily obtainable and cost-effective reactants, and the mitigation of adverse environmental impacts [124].

**Scheme 66:**
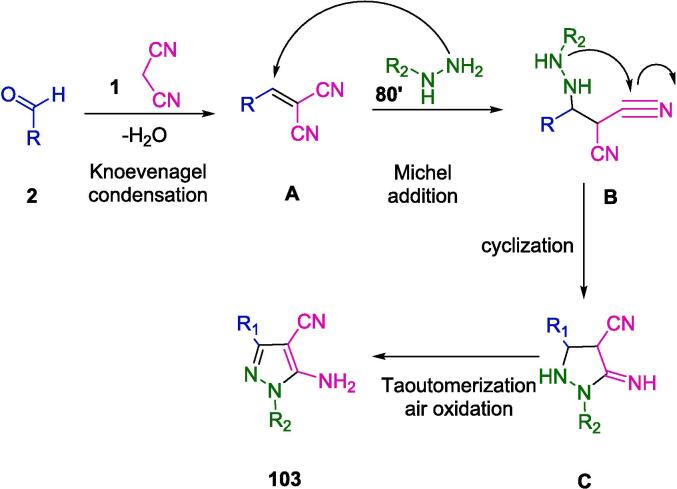
Plausible mechanism for synthesizing pyrazole derivatives.

A plausible mechanism for synthesizing pyrazole derivatives (**1 0 3**) is illustrated in Scheme 66, as described in the literature. The enolization of malononitrile (**1**) and the increased nucleophilicity of methylene carbon are facilitated by hydrogen bonding between PEG 400 and water. Through Knoevenagel condensation and Michael's addition, intermediates (**A)** and (**B)** are formed. In this catalyst-free system, phenyl hydrazine (**80**′) serves as both a Bronsted base and a nucleophile. The final product (**1 0 3**) is obtained by tautomerizing, and aromatizing intermediate (**C**).

**Scheme 67:**
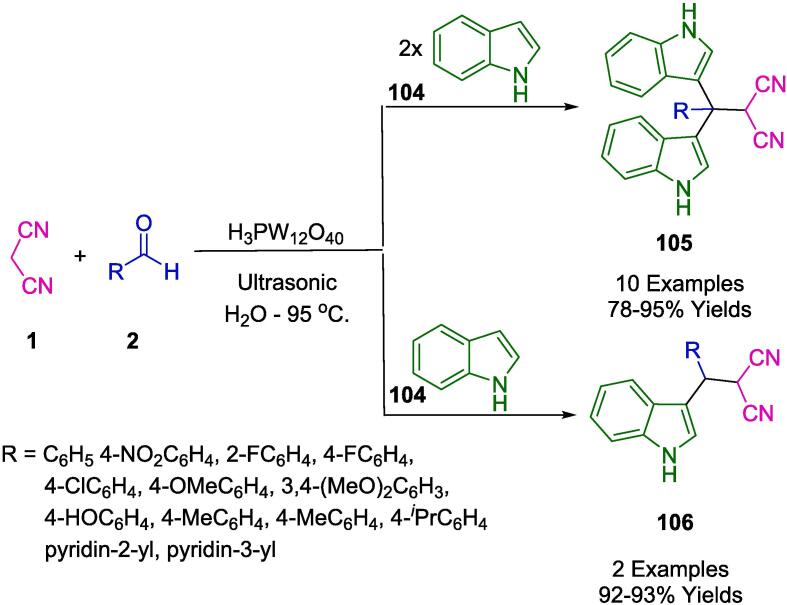
Synthesis of bis(indole) derivatives.

Bis(indole) motifs have garnered considerable interest as pivotal precursors in the generation of several compounds with pronounced biological activity [125], [126]. The research team led by Amrollahi innovated a streamlined and green strategy for the synthesis of these multifunctional compounds (**105**, **106**), using H_3_PW_12_O_40_ (Scheme 67). This methodology, which is catalyzed by ultrasonic waves, involves a one-pot condensation process that unites indole (**1 0 4**), aldehydes (**2**), and malononitrile (**1**), with 12-tungstophosphoric acid serving as the reaction medium, facilitated by ultrasonic irradiation in an aqueous environment. The process is characterized by its experimental simplicity, rapidity of reaction, and high yield rates. Additionally, it boasts of eco-friendly attributes, notably the omission of hazardous catalysts and solvents, further underscoring its environmental viability [127].

**Scheme 68:**
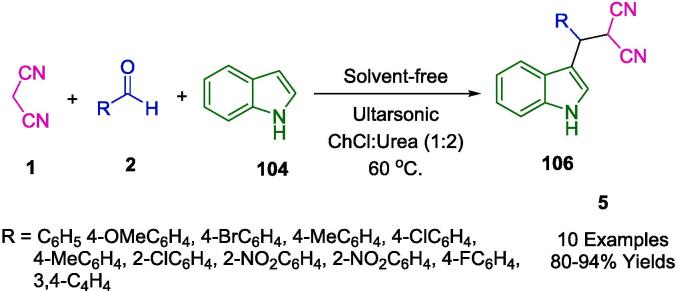
Synthesis of 3-substituted indole derivatives.

Deep eutectic solvents (DESs) are a class of ionic liquids (ILs) successfully implemented for many organic transformations [128]. Khandebharad and colleagues synthesized 3-substituted indoles (**1 0 6**), with the using a mixture of malononitrile (**1**), aromatic aldehyde (**2**), and indole (**1 0 4**) with 10 mol% of the DES prepared by the combination of ChCl:Urea (1:2) by utilizing the synergetic effect of ultrasound irradiation and the DES in green methods at 60 °C (Scheme 68). Their substitution patterns and potential for further modification make these indoles attractive as building blocks for organic synthesis [129].

## 7 Conclusion

The quest for sustainable synthetic methodologies that yield structurally diverse compounds with notable biological activities has been a significant research endeavor in contemporary chemistry. In alignment with this paradigm, the current review congregates a comprehensive analysis of multicomponent reactions (MCRs) that harness malononitrile as a key reactant, with a particular emphasis on the application of ultrasonic irradiation as a synthetic facilitator. The scope of applications for the synthetic procedures elucidated within this discourse underscores the criticality of this strategy, not only for its alignment with green chemistry principles but also for its ability to promote efficacious syntheses of heterocyclic compounds. These methods, underscored by their operational simplicity and environmental compatibility, stand at the forefront of chemical research. The review aims to catalyze further innovation by providing a foundation for the development of novel reactions that are not only benign by design but also capable of yielding compounds with potential therapeutic applications. The use of ultrasonic irradiation in the context of MCRs employing malononitrile is a testament to the ongoing evolution of green chemistry, and it embodies the collective aspiration to achieve sustainable industrial practices without compromising the pursuit of scientific advancement.

## CRediT authorship contribution statement

**Ramin Javahershenas:** Writing – review & editing. **Sahand Nikzat:** Resources.

## Declaration of competing interest

The authors declare that they have no known competing financial interests or personal relationships that could have appeared to influence the work reported in this paper.

## Data Availability

No data was used for the research described in the article.
